# Continuous timely monitoring of core temperature with two wearable devices in pediatric patients undergoing chemotherapy for cancer – a comparison study

**DOI:** 10.1007/s00520-024-08366-w

**Published:** 2024-02-24

**Authors:** Christa Koenig, Roland A. Ammann, Christine Schneider, Johanna Wyss, Jochen Roessler, Eva Brack

**Affiliations:** 1 Pediatric Hematology/Oncology, Department of Pediatrics, Inselspital, Bern University Hospital, University of Bern, CH-3010 Bern, Switzerland; 2 StatConsult Ammann, Burgdorf, Switzerland; 3 Faculty of Medicine, University of Bern, CH-3010 Bern, Switzerland; 4 Department of Pediatric Oncology and Hematology, University Children’s Hospital Basel (UKBB), University of Basel, Basel, Switzerland

**Keywords:** Temperature measurement, Fever, Continuous recording, Recording vital signs, Wearable device, Pediatric oncology, Supportive care

## Abstract

**Purpose:**

Pediatric patients with cancer often develop chemotherapy-induced fever in neutropenia (FN), requiring emergency broad-spectrum antibiotics. Continuous temperature monitoring can lead to earlier FN detection and therapy with improved outcomes. We aimed to compare the feasibility of continuous core temperature monitoring with timely data availability between two wearable devices (WDs) in pediatric oncology patients undergoing chemotherapy.

**Methods:**

In this prospective observational two-center study, 20 patients (median age: 8 years) undergoing chemotherapy simultaneously wore two WDs (CORE®, Everion®) for 14 days. The predefined goal was core temperature recorded in sufficient quality and available within ≤ 30 min during ≥ 18/24 h for ≥ 7/14 days in more than 15 patients.

**Results:**

More patients reached the goal with CORE® (*n* = 13) versus Everion® (*n* = 3) (difference, 50% *p* < 0.001). After correcting for the transmission bottleneck caused by two WDs transmitting via one gateway, these numbers increased (*n* = 15 versus *n* = 14; difference, 5%; *p* = 0.69). CORE® measurements corresponded better to ear temperatures (*n* = 528; mean bias, − 0.07 °C; mean absolute difference, 0.35 °C) than Everion® measurements (*n* = 532; − 1.06 °C; 1.10 °C). Acceptance rates for the WDs were 95% for CORE® and 89% for Everion®.

**Conclusion:**

The CORE® fulfilled the predefined feasibility criterion (15 of 20 patients) after correction for transmission bottleneck, and the Everion® nearly fulfilled it. Continuous core temperature recording of good quality and with timely data availability was feasible from preschool to adolescent patients undergoing chemotherapy for cancer. These results encourage the design of randomized controlled trials on continuously monitored core temperature in pediatric patients.

Trial registration.

ClinicalTrials.gov (NCT04914702) on June 7, 2021.

**Supplementary Information:**

The online version contains supplementary material available at 10.1007/s00520-024-08366-w.

## Introduction

Fever in chemotherapy-induced neutropenia (FN) is a frequent, potentially life-threatening complication in pediatric patients with cancer. During neutropenia, fever is often the first and only clinically detectable sign of infection. Therefore, FN is treated with emergency hospitalization and empirical intravenous broad-spectrum antibiotics [1]. Time to administration of antibiotics (TTA) is frequently used as a quality of care measure [2] because of a possible association of longer TTA with worse clinical outcomes [3]. Normally, temperature is only measured when fever is suspected. Correspondingly, the majority of FN episodes are diagnosed at temperatures above the predefined fever limit [4], which may lead to delayed therapy and worse outcomes. Furthermore, high fever is a risk factor for bacteremia [5, 6].

Wearable devices (WDs) are increasingly used to continuously monitor vital signs in different settings. WD-based continuous temperature monitoring with timely data accessibility may lead to earlier diagnosis, shorter TTA, and thus ultimately to improved outcomes in FN. Combined with other continuously measured vital signs, patterns predicting imminent fever or infection might additionally be detectable, further reducing TTA.

In pediatric patients with cancer, the feasibility of continuous temperature monitoring has been studied during hospitalization [7] and at home [8] using “stick-on” patches. A recently published case series describes three fever episodes in which fever was either detected earlier or only by such patches and consequently led the patients to seek medical attention [9]. In our previous study (NCT04134429), we showed that with the Everion®, continuous recording of vital signs in pediatric patients is possible over a wide age range [10]. However, the predefined feasibility criterion was missed, mainly due to non-compliance plus missing automated transmission rebooting after technical failures, thus independent of the WD assessed [10].

The primary aim of this study was to compare two different WDs regarding the feasibility of continuous core temperature recording of good quality with timely data accessibility in pediatric patients undergoing chemotherapy for cancer. Secondary feasibility-related study aims were (1) to compare feasibility for other vital signs recorded by the Everion®, (2) to compare feasibility disregarding time delay in data accessibility, (3) to assess the impact of the improved study setting on compliance using published Everion® data for comparison, and (4) to explore patient satisfaction, user-friendliness and side effects of the WDs, and investigator efforts. An additional secondary aim was (5) to compare the WDs regarding the agreement of continuously recorded core temperature measurements and discrete ear measurements. A tertiary aim was to explore patterns of vital signs recorded before the onset of fever or infection.

## Methods

### Study design and participants

For this prospective two-center observational study, patients with any malignancy aged 1 month to 17.99 years were screened for eligibility and recruited by the investigators (EB, CK, CS, JW). Inclusion criteria were treatment with myelosuppressive chemotherapy for any malignancy expected to last ≥ 1 month at the time of recruitment or with ≥ 1 cycle of myeloablative therapy before autologous/allogeneic hematopoietic stem cell transplantation. Patients were excluded when they had local skin disease prohibiting wearing WDs. Prior to study entry, patients, if able to judge, and their legal guardians gave written informed consent (IC). Participants could choose to wear both WDs in parallel, consecutively, or only one WD. All participants additionally gave non-mandatory IC for the publication of coded study data. The protocol was registered at www.clinicaltrials.gov (NCT04914702) and was approved by the local Ethics Committee (Kantonale Ethikkommission Bern, BASEC-No.: 2021–00967) prior to patient recruitment. The study was conducted in accordance with the Declaration of Helsinki [11] and the principles of Good Clinical Practice [12].

### Wearable devices and data management

**Fig. 1:**
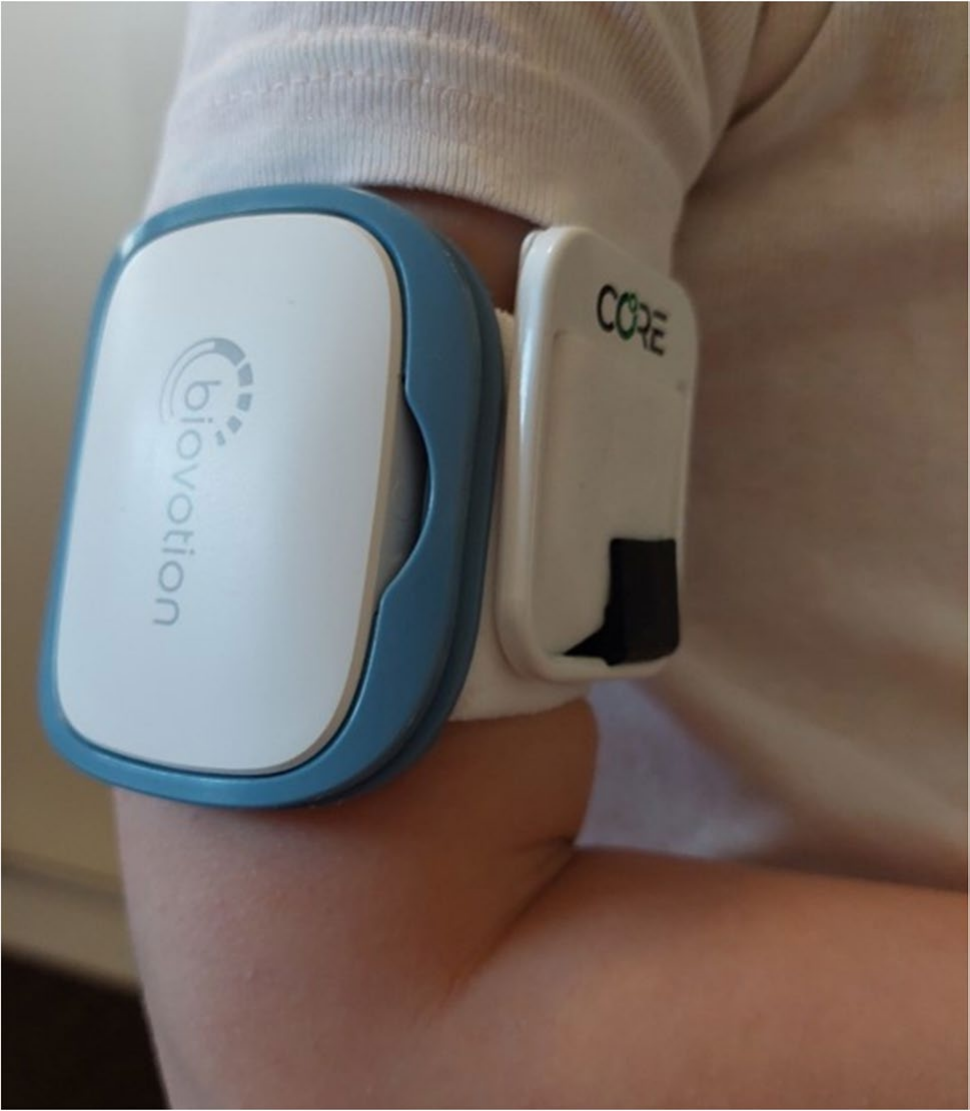
CORE® and Everion® worn on the upper arm in a five-year-old child. Displayed are the Everion® (left) and the CORE® (right), both worn together on one armband on the right upper arm of a five-year-old child. The CORE® is secured with a small black clip, which prevents it from disattachment from the armband. Parents gave informed consent for the publication of this photography

Two different WDs were used, the CORE® by greenTEG [13] and the Everion® VSM-1 by Biovotion (now Biofourmis) [14] (Fig. 1). Both WDs monitor the core temperature with its quality score once per minute. The Everion® additionally monitors further vital signs and calculated measures. See Online resource Text S1 for technical details. As battery charging for the Everion® takes several hours per day, patients received two Everions® and one CORE® (CORE ® daily battery charging of 30 min sufficient).

Both WDs transmitted data via Bluetooth® and a custom-built transportable gateway to a cloud-based dashboard, accessible for the research team only. To avoid influences on clinical decisions, the treating staff had no access to the vital signs. Parents and, when applicable, patients themselves received a link to their patient-specific continuously updated dashboard data on core temperature (CORE®), heart rate (Everion®), plus battery and transmission status (both WDs). This information was supplied with three aims: first, to enhance WD wearing compliance; second, to optimize battery recharging; and third, to avoid using the publicly available CORE® app, which interferes with data transmission from CORE® to the dashboard.

The entire data processing (transport and storage) was encrypted by Leitwert. For confidentiality, Leitwert had signed a data processing agreement.

Data was retrieved by the research team from the dashboard as csv files using json scripts provided by Leitwert and then imported into R [15]. Data were irreversibly deleted from the cloud-based dashboard after the completion of analysis and storage of WD data in a local research database. Non-WD data was collected on paper clinical research forms (CRFs) and stored using REDCap electronic data capture tools [16].

### Procedures

Participants received one CORE®, two Everions®, one gateway, and the respective chargers. Participants were instructed in WD and gateway handling and to wear the WDs during the entire 14-day study period. WDs were placed using size-specific elastic bands on the upper arm or leg (Fig. 1). Participants were instructed to measure ear temperature (Braun ThermoScan 7, provided for clinical routine use [17]) at least twice daily plus when clinically indicated and to note results and times on paper CRFs. Results of clinically indicated ear temperature measurements during hospitalization or outpatient visits were retrieved from charts.

Participants were encouraged to call the study center for technical issues or questions. The investigators (CK and EB) did daily data checks on the dashboard and gave daily feedbacks on wearing and data quality by either text message, e-mail, or phone call, as chosen by the participant. They discussed possible reasons for non-recording or insufficient data quality and gave corresponding instructions. Non-compliance to wear the WDs did not lead to exclusion from the study. A follow-up interview within three days after the study ended assessed the acceptability and usability of the WDs.

### Outcomes

The primary outcome was defined to register core temperature with a WD of sufficient data quality (CORE® ≥ 2, quality score 1 (lowest) to 4 (best), Everion® ≥ 50, quality score 0 (lowest) to 100 (best)) and with timely data accessibility on the dashboard (≤ 30 min after recording), during ≥ 18/24 h per day, on ≥ 7 of 14 days. The predefined criterion to claim feasibility was ≥ 15 out of 20 patients, fulfilling this patient-level outcome.

Secondary outcomes were comparable to the primary outcome (secondary aim 1), plus disregarding time delays (secondary aims 2 and 3). For secondary aim 4, outcome parameters matched the respective aim. For secondary aim 5, the difference between discrete ear temperature measurements and the corresponding continuously recorded core temperature was studied.

### Statistical analysis

A sample size of 20 patients per WD, including at least four patients below six years, was chosen, which enables to identify ≥ 95% of usability problems [18]. A formal sample size calculation was not performed. A posteriori power calculation revealed a power of 86% to detect a difference of 40% in the primary outcome between the two WDs, assuming a 50% probability of discordant pairs [19].

The per-second WD data was reformatted pseudo-randomly into per-minute data by selecting every 60th line for analysis. Correspondingly, times in the “Results” section were calculated in minutes and rounded to hours for display. Recorded data was categorized for data quality (at least sufficient, poor, no data recorded) and for the time delay (timely, i.e., arrival on dashboard ≤ 30 min after measurement, versus delayed). To eliminate the effects of the data transmission bottleneck due to large data volumes from two WDs transmitting via one gateway, simulated time delays corrected for this bottleneck effect were calculated retrospectively; see Online resource Text S2.

To assess the agreement of continuously recorded core temperatures with discrete ear measurements, Bland–Altman types of analyses and figures were used [20], including two exploratory analyses for the CORE®: First, core temperatures were recalculated offline by greenTEG using an experimental algorithm optimized in adults for arm positioning of the CORE®. Second, data-driven first- and second-order corrections were calculated based on linear mixed analysis regression of Bland–Altman type data, with a random intercept per patient, aiming to minimize fixed effect intercept and coefficients.

The two WDs were compared using tests of exact confidence intervals (CI) for differences of proportions for related or unrelated binomials, where applicable [20].

For the detection of specific patterns preceding fever or infections, vital signs were explored graphically. *P*-values < 0.05 were considered statistically significant. Version 4.1.1 of the R software [15] was used for data description and asymptotic analyses, and StatXact 10 [19] was used for exact analyses and power analysis.

## Results

### Patients

In two Swiss university-based pediatric hematology/oncology centers, 65 patients were consecutively screened for eligibility from September 2021 to March 2022. Seven did not meet inclusion criteria, 30 were asked for IC, while 28 were not asked after reaching the predefined patient number (Online resource Fig. S1 and Table S1). Twenty patients (median age 8 years; range: 2 to 17 years; 4 patients < 6 years), all opting to wear both WDs in parallel, were included in the study, 16 in Bern and 4 in Basel. Distribution of age, gender, type of malignancy in patients screened, not asked for IC, refusing IC, and those included in the study were comparable (Online resource Table S1). One patient withdrew IC before the study started, one on the first, and one on the fourth day. For all feasibility analyses, their results remained in the study. Potential data were thus available for 243 days, corresponding to 5832 h (87% of total 280*24 = 6720 h) for both WDs. Patients were hospitalized on 62 days, had outpatient visits on 17 days, and remained at home for 164 days. The WDs were worn on the upper arm by 19 patients and on the upper leg by one toddler of three years (patient 40).

### Primary outcome, uncorrected, both WDs

**Fig. 2:**
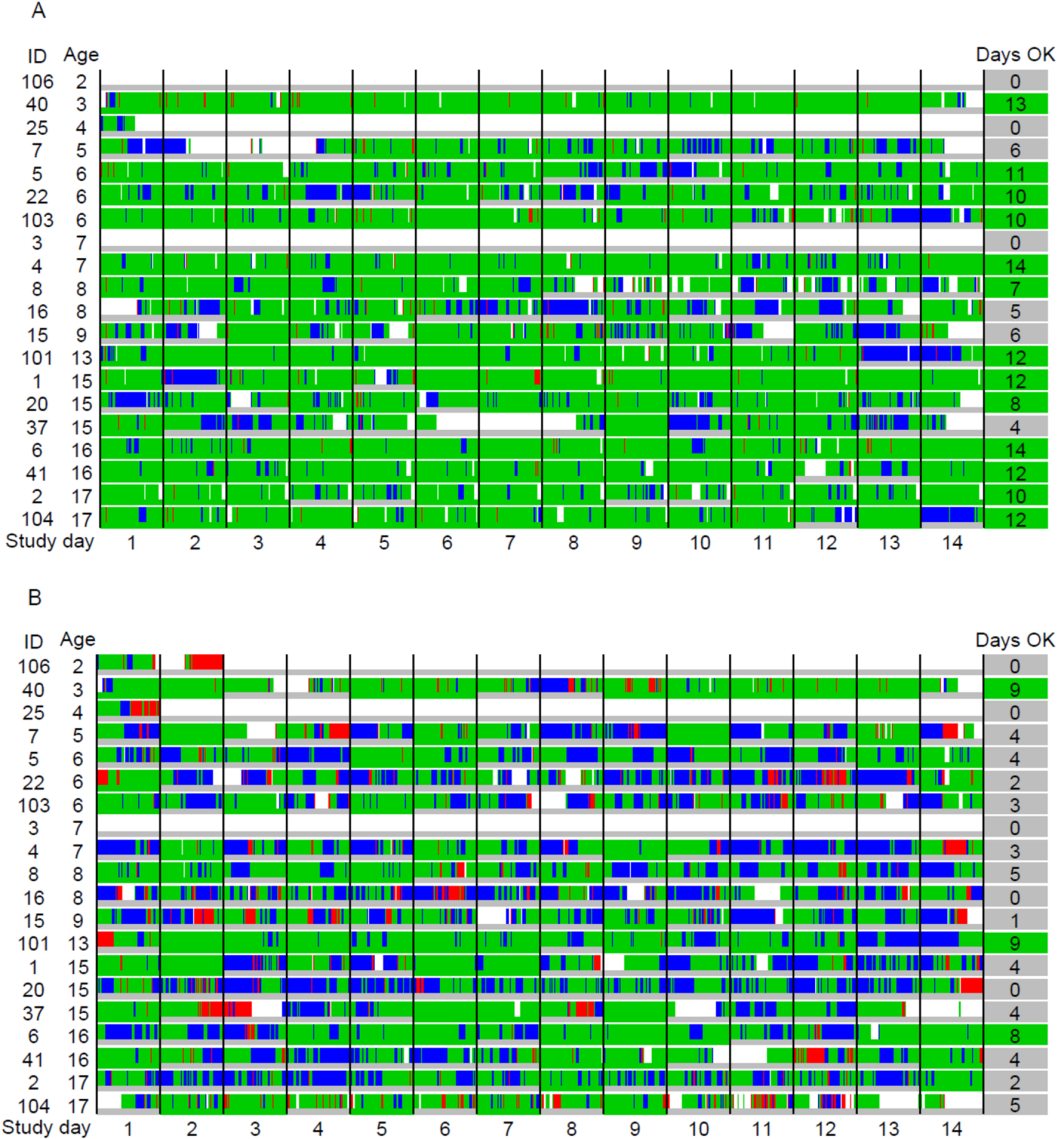
Primary outcome measure – recording of core temperature with the CORE® (**A**) and the Everion® (**B**). Green: at least sufficient quality of core temperature, arriving on the dashboard within ≤ 30 min. Blue: at least sufficient quality of core temperature, arriving on the dashboard after > 30 min. Red: poor data quality. White: no data recorded; days ok: number of days with ≥ 18 h at least sufficient data quality arriving on the dashboard within ≤ 30 min per patient; ID: patient ID

The CORE® recorded data on 4945 (74%) of 6720 study hours. Data arrived on the dashboard with a median delay of 2 min (IQR, 1 to 7). Data arrived timely during 4213 h (63%). Core temperature quality was at least sufficient during 4873 h (73%). The primary outcome measure was fulfilled during 4148 h (62%) and for ≥ 18/24 h per day in 166 days (59% of the total 280 study days). This corresponded to a median of 10 days per patient (range, 0 to 14) and to 13 of 20 patients, aged from 3 to 17 years, fulfilling the predefined patient-level goal (Table 1, Fig. 2).

**Table 1:** Comparison of CORE® versus Everion®: feasibility of continuous timely core temperature monitoring (primary outcome)

	Primary criterion fulfilled: hours^a^		Primary criterion fulfilled: Days^b^			Primary criterion fulfilled: Patients^c^		
	CORE®, hours (%)^d^	Everion®, hours (%)^d^	CORE®, days (%)^e^ (95% CI)	Everion®, days (%)^e^ (95% CI)	Comparison difference (95% CI), *p*-value	CORE® patients (%)^f^ (95% CI)	Everion® patients (%)^f^ (95% CI)	Comparison difference (95% CI), *p*-value
Uncorrected time delay	4148 (62%)	3349 (50%)	166 (59%) (53 to 65%)	67 (24%) (19 to 29%)	35% (29 to 42%), *p* < 0.001	13 (65%) (41 to 84%)	3 (15%) (4 to 39%)	50% (24 to 73%), *p* < 0.001
Time delay corrected for transmission bottleneck	4492 (67%)	4577 (68%)	173 (62%) (56 to 67%)	170 (61%) (55 to 66%)	1% (− 6 to 4%), *p* = 0.75	15 (75%) (51 to 90%)	14 (70%) (46 to 88%)	5% (− 18 to 23%), *p* = 0.69
Disregarding time delay	4873 (73%)	5056 (75%)	210 (75%) (69 to 80%)	198 (71%) (65 to 76%)	4% (− 1 to 9%), *p* = 0.097	17 (85%) (61 to 96%)	16 (80%) (55 to 93%)	5% (− 13 to 25%), *p* = 0.54

^a^Defined as at least sufficient quality of core temperature, arriving on the dashboard within ≤ 30 min

^b^Defined as at least sufficient quality of core temperature, arriving on the dashboard within ≤ 30 min, during ≥ 18 h per day

^c^Defined as at least the sufficient quality of core temperature, arriving on the dashboard within ≤ 30 min during ≥ 18 h per day on ≥ 7 days

^d^Proportion of the total study duration, 6720 h (20 patients * 14 days * 24 h per day)

^e^Proportion of the total study duration, 280 days (20 patients * 14 days)

^f^Proportion of the total number of patients, 20

The Everion® recorded data on 5360 h (80%). Data arrived on the dashboard with a median delay of 5 min (IQR, 1 to 68). Data arrived timely during 3556 h (53%). Core temperature quality was at least sufficient during 5056 h (75%). The primary outcome measure was fulfilled during 3349 h (50%) and for ≥ 18/24 h per day in 67 days (24%). This corresponded to a median of 3 days per patient (range, 0 to 9) and to 3 of 20 patients aged from 3 to 16 years, fulfilling the predefined patient-level goal (Table 1, Fig. 2).

The predefined criterion to claim feasibility was thus not fulfilled for both WDs. The CORE® was inferior to the Everion® regarding time with any data available. The CORE® was superior to the Everion® regarding the primary outcome on all levels studied, i.e., study hours, study days, and patients (Table 1).

### Primary outcome, corrected for data transmission bottleneck, both WDs

After correction for the bottleneck because of two WDs competing for data transmission on a single gateway, the median time delay decreased for both WDs, i.e., from 2 to 0 min (IQR, 0 to 1) for the CORE® and from 5 to 0 min (IQR, 0 to 2) for the Everion®. Correspondingly, the primary outcome fulfillment increased to 4492 (67%) hours to 173 (62%) days to 15 patients for the CORE® and to 4577 (68%) hours to 170 (61%) days to 14 patients for the Everion®.

After this correction, the predefined criterion to claim feasibility was thus fulfilled for the CORE® and nearly fulfilled for the Everion® (Online resource Fig. S2). The CORE® was comparable to the Everion® regarding the primary outcome on all levels studied, i.e., study hours, study days, and patients (Table 1).

### Quality of core temperature measurement disregarding time delay, both WDs

Disregarding time delay, the CORE® recorded core temperature in at least sufficient quality during 4873 (73%) h, during ≥ 18/24 h per day on 210 (75%) days, and during ≥ 18/24 h on ≥ 7 days in 17 of 20 patients. The corresponding results for the Everion® were 5056 (75%) h, 198 (71%) days, and 16 patients. Thus, when disregarding time delay, the core temperature feasibility measures were comparable in both WDs (Table 1).

### Further vital signs and measures, Everion® only

Feasibility results for further vital signs and measures of the Everion®, both applying and disregarding the time delay criterion, are displayed in Table 2.

**Table 2:** Secondary feasibility outcomes of vital signs recorded with the Everion®

	Primary criterion fulfilled: hours^a^		Primary criterion fulfilled: days^b^		Primary criterion fulfilled: Patients^c^	
	With time delay, hours (%)^d^	Disregarding time delay, hours (%)^d^	With time delay, days (%)^e^ (95% CI)	Disregarding time delay, days (%)^e^ (95% CI)	With time delay patients (%)^f^ (95% CI)	Disregarding time delay patients (%)^f^ (95% CI)
Heart rate	3342 (50%)	5046 (75%)	67 (24%) (19 to 29%)	198 (71%) (65 to 76%)	3 (15%) (4 to 39%)	16 (80%) (56 to 93%)
Heart rate variability	2123 (32%)	3134 (47%)	8 (3%) (1 to 6%)	28 (10%) (7 to 14%)	0 (0%) (0 to 20%)	1 (5%) (0 to 27%)
Respiration rate	3174 (47%)	4741 (70%)	58 (21%) (16 to 26%)	188 (67%) (61 to 73%)	2 (10%) (2 to 33%)	15 (75%) (51 to 90%)

^a^Defined as at least sufficient quality of core temperature, arriving on the dashboard within ≤ 30 min

^b^Defined as at least sufficient quality of core temperature, arriving on the dashboard within ≤ 30 min, during ≥ 18 h per day

^c^Defined as at least the sufficient quality of core temperature, arriving on the dashboard within ≤ 30 min during ≥ 18 h per day on ≥ 7 days

^d^Proportion of the total study duration, 6720 h (20 patients * 14 days * 24 h per day)

^e^Proportion of the total study duration, 280 days (20 patients * 14 days)

^f^Proportion of the total number of patients, 20

### Impact of improved study setting, Everion® only

To assess the impact of study setting improvements, the primary outcome of the predecessor study, NCT04134429, was at least sufficient heart rate data quality during ≥ 18/24 h on ≥ 7 consecutive days. Its fulfillment, mainly reflecting Everion® wearing time, increased from 3992 to 5045 study hours (59% vs. 75%), from 142 to 198 days (51% vs. 71%; difference, 20%; 95% CI, 12 to 28; *p* < 0.001), and from 6 to 11 patients (difference, 25%; 95% CI, − 7 to 53; *p* = 0.13). (Online resource Fig. S3).

### Patient and parent satisfaction, user-friendliness, and side effects

The follow-up questionnaire was answered by 19 participants by parents (*n* = 10), patients (*n* = 2), or both (*n* = 7). Of these, 18 judged the CORE® and 17 the Everion® to be suitable to record core temperature in children undergoing chemotherapy. No major problems regarding WD size, comfort, charging, and time needed for use were reported (Online resource Table S2). The gateway was perceived as too heavy (*n* = 7) and tedious to carry (*n* = 13), while handling was no problem (*n* = 18); 11 participants reported continuous data feedback as motivating, while 3 reported that this sometimes led to uncertainty; 11 participants reported daily feedbacks as motivating, and none experienced them as disturbing (Online resource Table S2).

Four participants reported occasional sweating underneath the WDs/armband, leading to slightly irritated skin without intervention needed in one patient. No further side effects were reported.

### Effort for the investigators

About 82 h of investigator’s efforts were recorded for inclusion, first instruction, daily data checks, and feedbacks, plus additional contacts for problems, for a total of 280 study days in 20 patients. Participants’ inclusion and instruction took approximately 2 h per patient (a total of 40 h). Daily data checks and feedbacks to all patients took about 10 min per participant and study day (total of 40 h, Online resource Table S3). There were 40 additional contacts for problems with a cumulative duration of 2 h, equally initiated by investigators and participants (20 each, Online resource Table S3).

### Comparison of continuously recorded core temperature with discrete ear temperature measurements

**Fig. 3:**
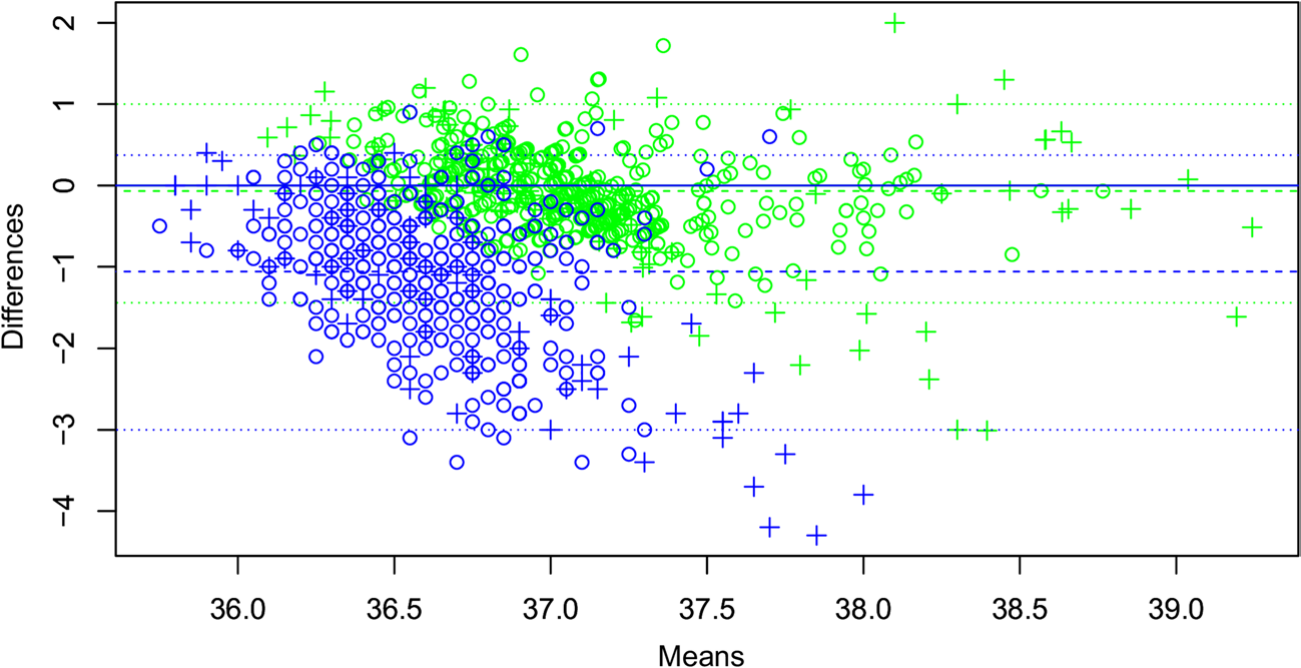
Bland–Altman plot showing means (*x*-axis) and differences (*y*-axis) between discrete ear temperature measurements and corresponding recordings of the core temperature of the CORE® (green) and the Everion® (blue). Crosses indicate measurements in patient 40, wearing devices on the upper leg; circles indicate measurements in all other patients, wearing devices on the upper arm

In total, 650 discrete measurements of ear temperature were noted (median per patient, 30; range, 0 to 108) with 524 CORE® and 532 Everion® coincident core temperature measurements analyzable. The CORE® consistently outperformed the Everion®: The mean difference against ear temperature was − 0.07 °C versus − 1.06 °C, the mean absolute difference was 0.35 °C versus 1.10 °C, and the width of the temperature range covering 95% of data points in the Bland-Altman diagram was 2.44 °C (− 1.44 to 1.00) versus 3.37 °C (− 3.00 to 0.37; Fig. 3).

### Exploration for specific patterns preceding fever or infections

Two episodes of fever were recorded in patients 40 and 41, both occurring during hospitalization. Visual exploration of the recorded vital signs did not show evident patterns (Online resource Fig. S4).

## Discussion

The predefined criterion to claim feasibility, i.e., ≥ 15 of 20 patients fulfilling the patient-level goal for core temperature measurement, was almost reached for the CORE® (13 of 20 patients) and clearly missed for the Everion® (3 of 20 patients). This significant difference was mainly due to longer time delays of the Everion®, reflecting its shorter Bluetooth® range, while core temperature quality and time of wearing were good and comparable for both WDs.

The suboptimal setting of two WDs competing for data transmission on a single gateway led to a strong transmission bottleneck and relevantly increased time delays, mainly for the Everion® and less for the CORE®. After correcting time delays by simulating a bottleneck-free setting of one gateway per WD, the predefined feasibility criterion was fulfilled for the CORE® and nearly fulfilled for the Everion® without significant differences between the two WDs. This simulated bottleneck-free setting was not defined in the study protocol because the bottleneck was not anticipated. It does, however, better reflect the setting of a study critically relying on timely data availability, e.g., a randomized controlled trial (RCT) with an intervention guided by continuously measured core temperature. Such a transmission bottleneck-free setting is easily reached by using one WD per gateway, by reducing the transmitted data volume with, e.g., per-minute instead of per-second data, or by a software-implemented massive transmission advantage of the WD relevant for the RCT part of the study over, e.g., the WD relevant only for offline pattern detection.

Compared to the predecessor study on Everion® feasibility (NCT 04134429 [10]), the study setting was improved by feedbacks plus a more robust transmission solution. Specifically, daily personal feedbacks, plus continuously available information on battery status, main results, and transmission delay of both WDs, aimed to increase compliance, i.e., wearing time of the WD. Replacement of a smartphone transmission app by an automatically rebooting dedicated physical transmission gateway aimed to reduce transmission delays and data loss if the internal WD storage capacity was exceeded. These changes led to significant improvements in Everion® wearing time and thus of the NCT 04134429 primary outcome. All three improvements merit to be implemented in future studies, relying on the timely availability of continuously recorded core temperature or other vital signs, irrespective of the specific WD used.

A large number of core temperatures continuously recorded by the two WDs could be compared with discrete ear temperature measurements, but only rarely for temperatures exceeding 38.0 °C. While there was no relevant bias for the CORE®, there was a strong bias, which became even stronger for high temperatures, for the Everion®. Ear temperature was used as the gold standard because it is used clinically, despite the fact that it is known not to optimally represent core temperature [21, 22]. This means that it remains unclear if the source of the differences found lies in the ear temperature measurements and/or in the WD measurements.

Participants’ satisfaction was good. The vast majority of participants considered both WDs to be suitable, easy, and comfortable to wear. No side effects except for sweating and slightly irritated skin were reported. The age range of patients fulfilling the patient-specific primary aim was wide. This implies that future clinical applications of both WDs for continuous and at-home recording of core temperature for the CORE® and of other vital signs for the Everion® can be envisaged from preschool children to adolescents. Infants were not studied here.

In children undergoing chemotherapy for cancer, several studies with different WDs have assessed physical activity [23–26], heart rate [27], and fever [9]. Along with our own predecessor study [10], no study reported timely data accessibility for continuously monitored vital signs. These studies, including ours, show however the potential of this technology, but their exploitation and resulting evidence currently are scarce, and further studies, including RCTs, are needed.

Our study has some limitations: (i) The simulated transmission bottleneck correction may reduce the validity of the corresponding results. (ii) WDs were studied for only 14 days, and results might differ when used for longer time periods. (iii) The non-standard placement of COREs® on extremities instead of the chest may have deteriorated core temperature measurements, although explorative offline analyses using an experimental upper arm algorithm did not relevantly change results. (iv) The role of medical staff in temperature monitoring was not assessed, so its influence remains unclear.

The major strengths of this study are the inclusion of an unbiased sample of children and adolescents undergoing chemotherapy for cancer, the additional assessment of data transmission times, and the evaluation of the impact of design improvements compared to a predecessor study [10]. The parallel assessment of two WDs, the broad assessment of feasibility aspects including participants’ opinions and effort for the investigators, allows a comprehensive judgment of feasibility to monitor vital signs in pediatric patients undergoing chemotherapy for cancer and a direct comparison of the WDs assessed.

In conclusion, continuous core temperature monitoring with good quality and timely data availability was feasible across a wide age range in pediatric patients undergoing chemotherapy for cancer. These results encourage the design of randomized controlled trials relying on continuously measured core temperatures available online in pediatric patients.

### Supplementary Information

Below is the link to the electronic supplementary material.

Supplementary file1 (PDF 3.41 MB)

## Data Availability

The data of this study will be made openly available on figshare at 10.6084/m9.figshare.22507342.

## References

[1] Lehrnbecher T, Robinson PD, Ammann RA et al (2023) Guideline for the management of fever and neutropenia in pediatric patients with cancer and hematopoietic cell transplantation recipients: 2023 Update. J Clin Oncol 20;41(9):1774–1785. 10.1200/JCO.22.02224 10.1200/JCO.22.02224 PMC10022858 36689694

[2] McCavit TL, Winick N (2012). Time-to-antibiotic administration as a quality of care measure in children with febrile neutropenia: a survey of pediatric oncology centers. Pediatr Blood Cancer.

[3] Koenig C, Schneider C, Morgan JE, Ammann RA, Sung L, Phillips B (2020). Association of time to antibiotics and clinical outcomes in patients with fever and neutropenia during chemotherapy for cancer: a systematic review. Support Care Cancer.

[4] Koenig C, Bodmer N, Agyeman PKA, et al (2020) 39.0°C versus 38.5°C ear temperature as fever limit in children with neutropenia undergoing chemotherapy for cancer: a multicentre, cluster-randomised, multiple-crossover, non-inferiority trial. Lancet Child Adolesc Health 4(7):495–502. 10.1016/S2352-4642(20)30092-4 10.1016/S2352-4642(20)30092-4 32497520

[5] Rackoff WR, Gonin R, Robinson C, Kreissman SG, Breitfeld PB (1996). Predicting the risk of bacteremia in children with fever and neutropenia. J Clin Oncol.

[6] Rosenman M, Madsen K, Hui S, Breitfeld PP (2002). Modeling administrative outcomes in fever and neutropenia: clinical variables significantly influence length of stay and hospital charges. J Pediatr Hematol Oncol.

[7] Sampson M, Hickey V, Huber J, Alonso PB, Davies SM, Dandoy CE (2019). Feasibility of continuous temperature monitoring in pediatric immunocompromised patients: a pilot study. Pediatr Blood Cancer.

[8] Kakarmath SS, de Redon E, Centi AJ, Palacholla R, Kvedar J, Jethwani K, Agboola S (2018). Assessing the usability of an automated continuous temperature monitoring device (iThermonitor) in pediatric patients: non-randomized pilot study. JMIR Pediatr Parent.

[9] Nessle CN, Flora C, Sandford E, Choi SW, Tewari M (2022). High-frequency temperature monitoring at home using a wearable device: a case series of early fever detection and antibiotic administration for febrile neutropenia with bacteremia. Pediatr Blood Cancer.

[10] Koenig C, Ammann RA, Kuehni CE, Roessler J, Brack E (2021). Continuous recording of vital signs with a wearable device in pediatric patients undergoing chemotherapy for cancer-an operational feasibility study. Support Care Cancer.

[11] World Medical Association (2013) Declaration of Helsinki. https://www.wma.net/policies-post/wma-declaration-of-helsinki-ethical-principles-for-medical-research-involving-human-subjects. Accessed 20 Apr 2023

[12] International Council for Harmonisation of Technical Requirements for Pharmaceuticals for Human Use (ICH) (2016) Guideline for Good Clinical Practice E6(R2) https://www.ich.org/page/efficacy-guidelines#6-2. Accessed 20 Apr 2023

[13] greenTEG, CORE. https://shop.greenteg.com/core-body-temperature-monitor. Accessed 19 Dec 2023

[14] Biofourmis AG. Everion monitor. https://support.biofourmis.com/hc/en-us/categories/201377109-Everion-Device. Accessed 20 Apr 2023

[15] R Core Team (2014) R: a language and environment for statistical computing. Vienna, Austria. http://www.R-project.org/

[16] Harris PA, Taylor R, Thielke R, Payne J, Gonzalez N, Conde JG (2009). Research electronic data capture (REDCap) - a metadata-driven methodology and workflow process for providing translational research informatics support. J Biomed Inform.

[17] Braun Thermoscan Ear Thermometer (IRT 6520, IRT 6020) https://de.scribd.com/document/347569924/Irt6020-6520-Westerneurope-Ownermanual-04mar14. Accessed 19 Dec 2023

[18] Faulkner L (2003). Beyond the five-user assumption: benefits of increased sample sizes in usability testing. Behav Res Methods Instrum Comput.

[19] Cytel Software, StatXact 10, Cambridge MA. https://www.cytel.com/. Accessed 19 Dec 2023

[20] Altman DG (1991). Practical statistics for medical research.

[21] Zhen C, Xia Z, Long L, Pu Y (2014). Accuracy of infrared ear thermometry in children: a meta-analysis and systematic review. Clin Pediatr (Phila).

[22] Twerenbold R, Zehnder A, Breidthardt T, Reichlin T, Reiter M, Schaub N, Bingisser R, Laifer G, Mueller C (2010). Limitations of infrared ear temperature measurement in clinical practice. Swiss Med Wkly.

[23] Braam KI, van Dijk-Lokkart EM, Kaspers GJL, Takken T, Huisman J, Bierings MB, Merks JHM, van de Heuvel-Eibrink MM, van Dulmen-den BE, Veening MA (2016). Cardiorespiratory fitness and physical activity in children with cancer. Support Care Cancer.

[24] Aznar S, Webster AL, San Juan AF (2006). Physical activity during treatment in children with leukemia: a pilot study. Appl Physiol Nutr Metab.

[25] Hooke MC, Gilchrist L, Tanner L, Hart N, Withycombe JS (2016). Use of a fitness tracker to promote physical activity in children with acute lymphoblastic leukemia. Pediatr Blood Cancer.

[26] Winter C, Muller C, Brandes M, Brinkmann A, Hoffmann C, Hardes J, Gosheger G, Boos J, Rosenbaum D (2009). Level of activity in children undergoing cancer treatment. Pediatr Blood Cancer.

[27] Vaughn J, Gollarahalli S, Shaw RJ, Docherty S, Yang Q, Malhotra C, Summers-Goeckerman E, Shah N (2020). Mobile health technology for pediatric symptom monitoring: a feasibility study. Nurs Res.

