# Supplementary material for: Continuous timely monitoring of core temperature with two wearable devices in pediatric patients undergoing chemotherapy for cancer – a comparison study
Source: Support Care Cancer. 2024 Feb 24;32(3):188. doi: 10.1007/s00520-024-08366-w (PMC10894150; doi:10.1007/s00520-024-08366-w)
Supplement: Supplementary file 1 — Supplementary file1 (PDF 3.41 MB) [file 520_2024_8366_MOESM1_ESM.pdf]

Supplementary material

Continuous timely monitoring of core temperature with two wearable devices in pediatric patients undergoing chemotherapy for cancer – a comparison study

Christa Koenig*, Roland A. Ammann*, Christine Schneider, Johanna Wyss, Jochen Roessler,

Eva Brack

*shared first authorship

Contents

Online Resource Text S1 – Technical details of wearable devices 2

Online Resource Text S2 - Simulating delay times corrected for data transmission bottleneck. 2

Online Resource Figure S1 - Flow chart patient recruitment 4

Online Resource Figure S2 - Primary outcome measure corrected for data transmission bottleneck – recording of core temperature with the CORE® (A) and the Everion® (B) 5

Online Resource Figure S3 – Impact of improved study setting on Everion® data 7

Online Resource Figure S4 – All signals, all patients and all study days 9

Online Resource Figure S5 – Transmission delays of CORE® and Everion® data 14

Online Resource Figure S6 – Data volume measurement in a section with non-delayed data transmission for CORE® and Everion® 15

Online Resource Figure S7– Transmission speed measurement in a section with continuous transmission of delayed CORE® data 16

Online Resource Figure S8 – Transmission speed measurement in a section with continuous transmission of delayed Everion® data 17

Online Resource Figure S9 – Transmission delays and simulated delays of CORE® and Everion® data 18

Online Resource Table S1 - Basic patient characteristics. 19

Online Resource Table S2 - Details on patient and parent satisfaction and user-friendliness. 20

Online Resource Table S3 - Details on feedbacks, additional contacts and problems identified. 21

Online Resource Table S4 – Signals recorded by Everion® and CORE®. 22

Online Resource Text S1 – Technical details of wearable devices

The Everion® (version, VSM-1; reference number, 910044; firmware version, 3.0.6) is CE-certified (CE 0123), medical device class IIa and FDA 510(k) exempt listed. It is light (40g), small (70x55x9 mm) and designed to be worn on the upper arm, see Figure 1. It records skin temperature once per second, and calculates core temperature once per minute, including its quality score (range, 0 to 100; sufficient quality, score ≥ 50). It additionally records heart rate, heart rate variability, respiration rate and further vital signs plus calculated measures, most of them once per second, some of them with their respective quality scores. In order to reduce energy consumption, the Everion® was set to mode «green only», which precludes from measuring oxygen saturation. We have previously shown that the quality of the oxygen saturation signal is poor in the majority of time [25].See Online Resource Table S4 for a full list of monitored signals.

The CORE® (lots GTG0013/17/18/19; firmware version, 0.3.15) is a commercially available consumer WD. It is not certified as a medical product and so no CE certificate is available. However the device meets the following EU conformity standards: 2014/53/EU Radio Equipment Directive (RED) and 2011/65/EU Restriction of Hazardous Substances (RoHS). It is lighter (12g) and smaller (50x40x 8 mm) than the Everion®. It is designed to be worn on the chest, either by an elastic strap or by skin-compatible adhesives. In this study, all but one patient wore it on the upper arm, see Figure 1. It measures skin temperature once per second, and calculates core temperature using an algorithm optimized in adults for chest positioning once per minute, including its quality score (range, 0 to 4; sufficient quality, score ≥ 2).

The data recorded and stored temporarily by the WDs were sent to a password protected cloud dashboard via a gateway. The gateway was custom built from commercially available components (Raspberry Pi 3B+ microcomputer from raspberry.org, uninterruptible power supply UPS HAT from waveshare.com equipped with 4 Li-ion batteries with a total capacity of 13000mAh, E3372h-320 GSM dongle from huawei.com) plus a Leitwert programmed BLED112 USB dongle for Bluetooth connection to the Everion®. All components were packed into a robust case, the entire gateway weighing around 500g. It run on proprietary gateway software by Leitwert (version 1.11.0, with the bgapi library, version 0.5.2, for the BLED112 dongle integrated), and connected to the WDs via Bluetooth® and to the dashboard via wireless LAN or the mobile net, without storing or processing the data. After shutdown by, e.g., inadvertent switching off, or battery exhaustion, the gateway re-booted automatically and was ready for data transmission within two minutes.

The dashboard was a GCP-ready server application hosted in Switzerland and specifically adapted to the needs of this study (Leitwert Dashboard, frontend version 3.7.12, backend version 3.7.5). To compensate for the wider range of the Bluetooth connection of the CORE®, the gateway configuration setting “default minimum synchronization interval” was increased to 120 seconds for the CORE® versus 60 seconds for the Everion®.

In the current setting, approximate charging times until full battery capacity were 3 hours for 18 hours recording in the Everion®, 3 hours for 120 hours (5 days) recording in the CORE®, and about 8 hours during function for 18 hours transmission in the gateway. The gateway was rechargeable during function thanks to the dual function capability of its power supply unit, while the Everion® and the CORE® had to be charged off body and thus off function. Correspondingly, the gateway was usually recharged some hours during every night in the sleeping room, the CORE® was usually recharged every day for about half an hour during, e.g, the time of bathing, and the two Everions® were alternatingly charged during night and during day, respectively.

Online Resource Text S2 - Simulating delay times corrected for data transmission bottleneck.

Retrospectively, the data transmission setting used here was suboptimal in two dimensions. First, per-second data were chosen for transmission, while only per-minute data were analysed; and second, the two WDs were transmitted by a single gateway, transmitting the entire data volume of one WD continuously before transmitting data of the other WD. This setting was good when both WDs were constantly within the Bluetooth range of the gateway, the transmitting WDs then changing every minute, with data transmission always being up-to-date. After long data transmission breaks, e.g., when the gateway had not been taken to the school, this setting led to a bottleneck in transmission capacity, with relevant additional transmission delays for one of the WDs, while the large volume of data of the other WD were transmitted. Frequently, the large data volume to be transmitted with a relatively slow speed led to additional delays of transmission for the WDs after transmission breaks. As an instructive example in patient 8, Online Resource Figure S5 shows such delays for both WDs: After a transmission break of around 300 min (study min 6530 to 6830), the CORE® has exclusive data transmission for around 10 minutes, leading to an additional delay of around 10 minutes for Everion® data measured on study min 6530 to 6850. Non-typically, transmission then switches to the Everion® for more than 20 min even before

complete transmission of the CORE® data. This leads to additionally delayed transmission of CORE® data for min 6530 to 6580, i.e., at the beginning of transmission break, and min 6830 to 6880, when transmission has been re-established for nearly an hour. This Figure displays as well the different transmission modes of the two WDs: The CORE® transmits last-in-first-out (LIFO), i.e. the data measured most recently are transmitted first, before older data. This is the reason of the longer delays of data measured at the beginning of the transmission break compared to more recent data. The Everion®, however, transmits first-in-first-out (FIFO), i.e. the data measured first, thus the oldest data, are transmitted before more recent data. These additional delays were corrected by simulating, in silico, a one gateway per WD setting.

Specifically, the per-second data resulted in large data volumes to be transmitted. The CORE® generated a data volume of 10.2 MB per day, corresponding to 425 kB per hour and 7 kB per minute measuring time, as assessed on study day 9 of patient 37, with 100% coverage (Online Resource Figure S6). The Everion® generated a data volume of 25.9 MB per day, corresponding to 1079 kB per hour and 18 kB per minute measuring time. The median data transmission speed was measured in 10 time periods with exclusive and continuous transmission per WD (Online Resource Figures S7 and S8). The median transmission speed of the CORE® was 30 min of data transmitted per minute, corresponding to 212 kB transmitted per min. The median transmission speed of the Everion® was 12 min of data transmitted per min, corresponding to 90 kB transmitted per min.

Simulating in silico a one gateway per WD setting, avoiding this transmission bottleneck, was based on LIFO transmission at a speed of 30 min data per 1 min transmission time for the CORE®, and on FIFO transmission at a speed of 12 min data per 1 min transmission time for the Everion®. When, in reality, one WD was transmitting, transmission was simulated additionally for the other WD. The effects of this simulation are displayed in Online Resource Figure S9 for the patient 8 example discussed above: For the CORE®, the additional delay for data measured at the beginning of the transmission break is eliminated. Of course, these data remain strongly delayed because of the long transmission break as such. From min 6580 to 6830, the simulated delays coincide with real delays because of the exclusive CORE® transmission time. The delays of CORE® data measured from min 6830 to 6870, due to the subsequent exclusive Everion® transmission time, however, are eliminated. For the Everion®, simulated delays of data measured in the entire transmission break time plus around 30 additional minutes are around 10 minutes lower than delays in reality. The different effects of LIFO versus FIFO transmission are as well visible in these simulation data.

Online Resource Figure S1 - Flow chart patient recruitment

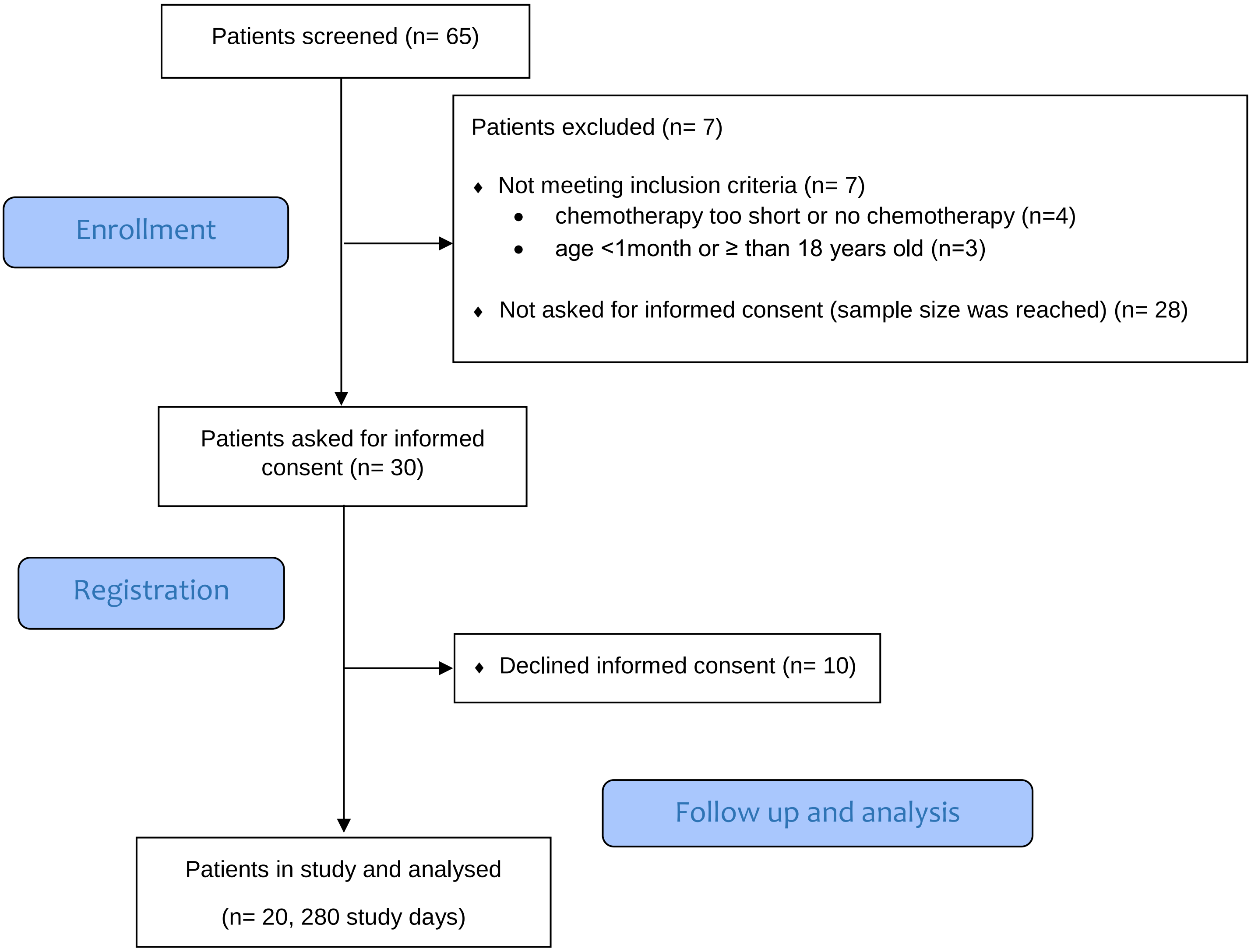

Online Resource Figure S2 - Primary outcome measure corrected for data transmission bottleneck – recording of core temperature with the CORE® (A) and the Everion® (B)

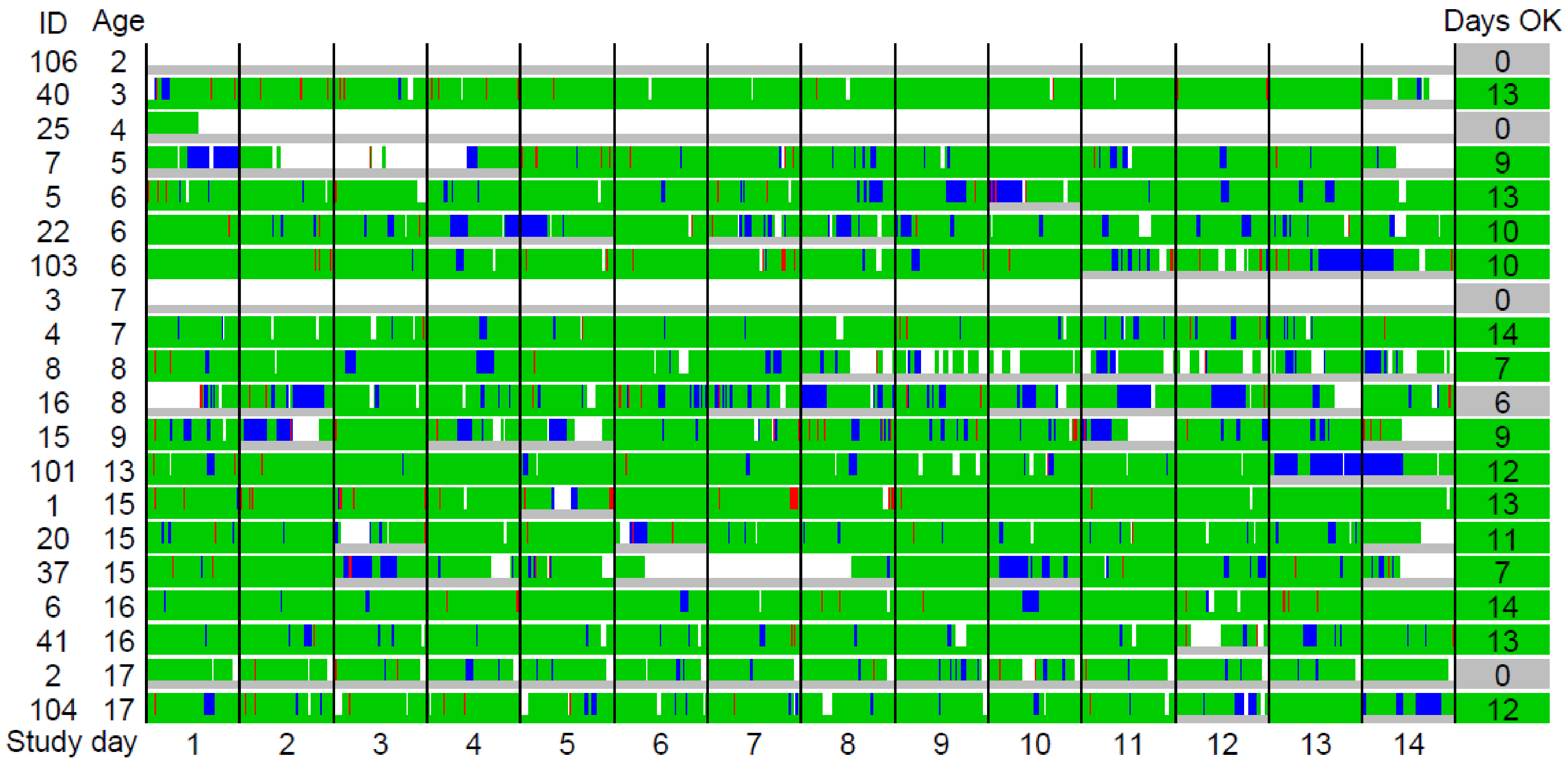

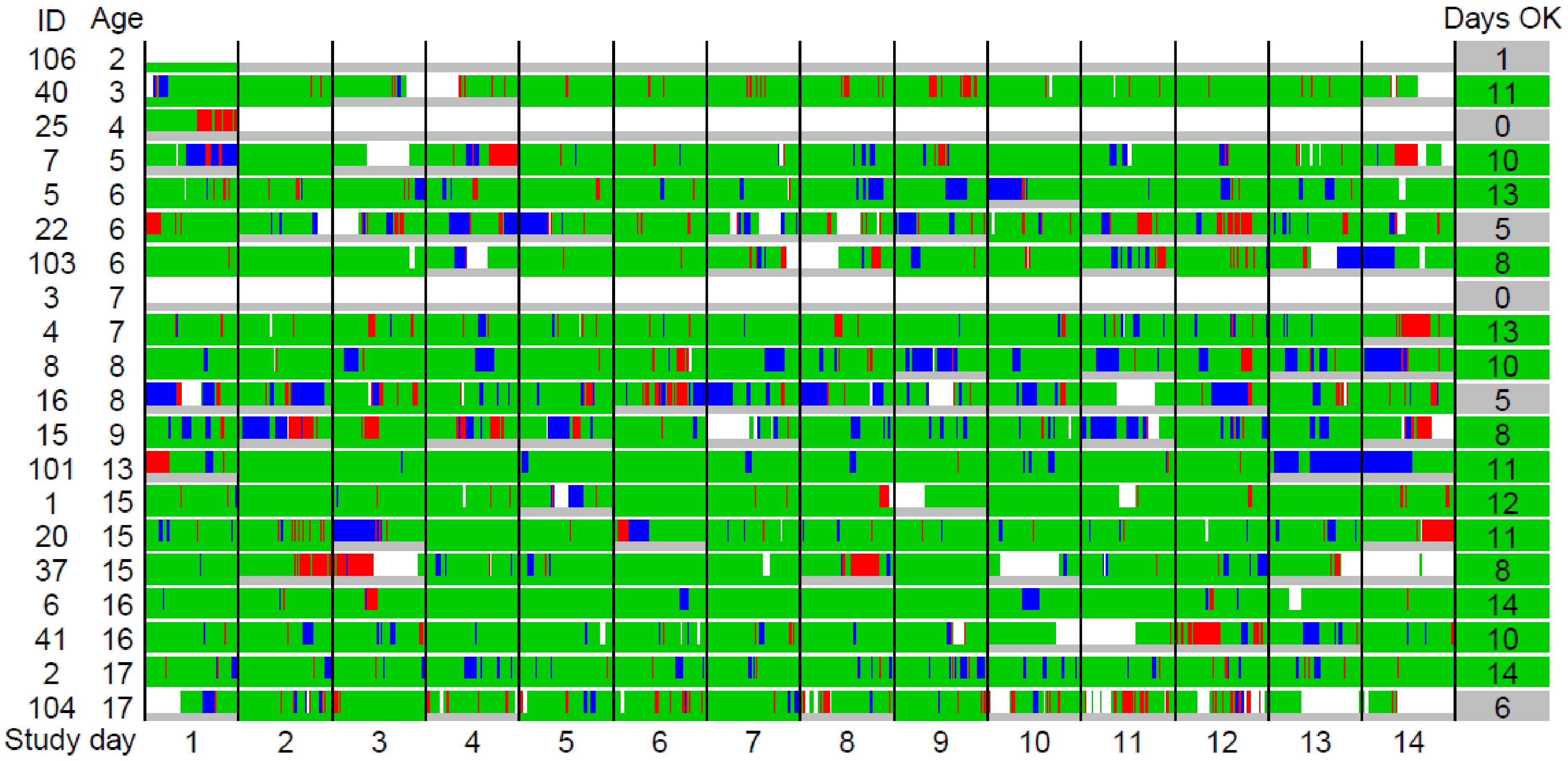

Displayed are the results of core temperature recording after correction for data transmission bottleneck, by the CORE® in A) the Everion® B).
Legend: green, at least sufficient data quality of core temperature, arriving on dashboard within ≤30min; blue, at least sufficient quality of core temperature, arriving on dashboard after >30min; red: poor data quality; white: no data recorded; Days ok: number of days with ≥ 18h at least sufficient data quality arriving on dashboard within ≤30min per patient; ID: patient ID

Online Resource Figure S3 – Impact of improved study setting on Everion® data

A

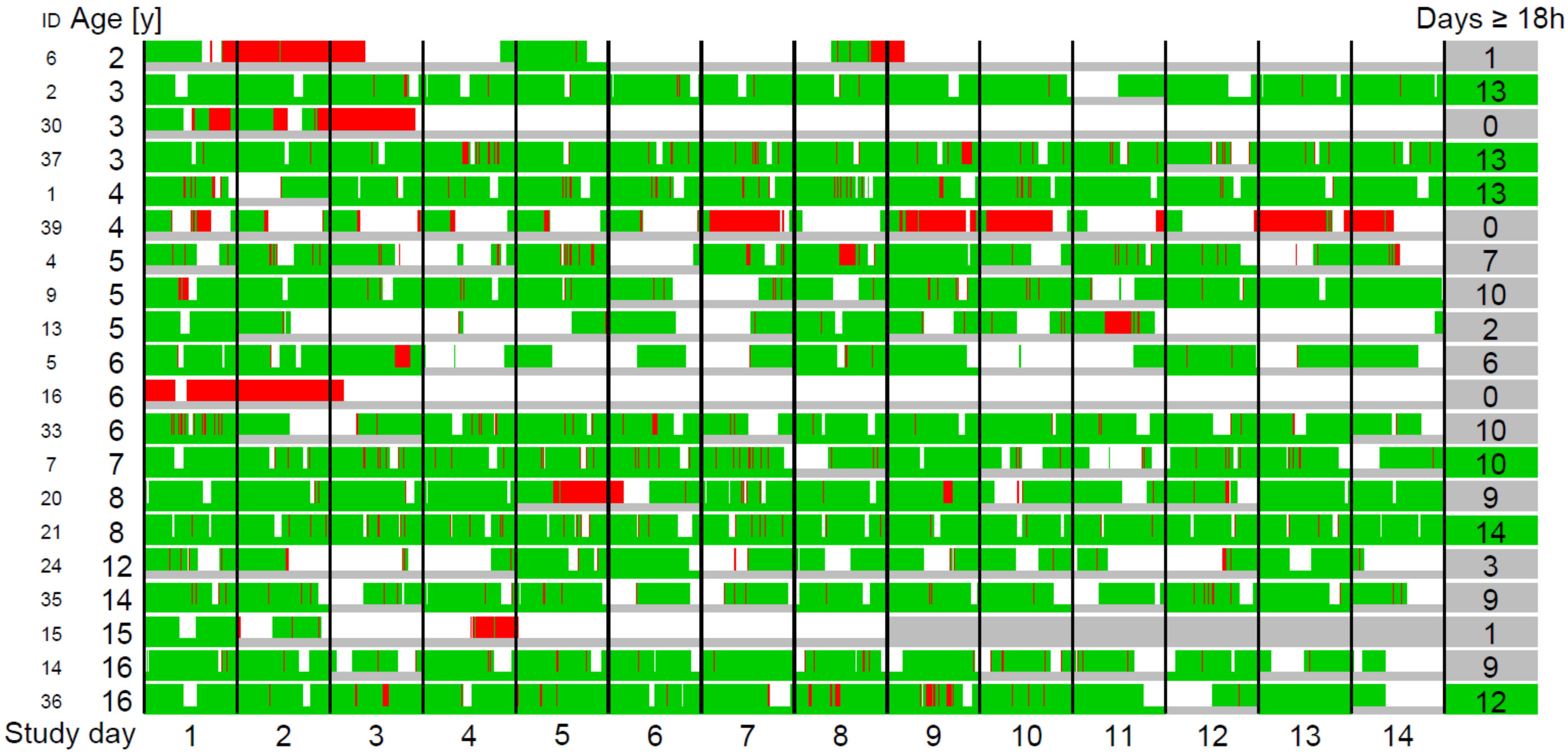

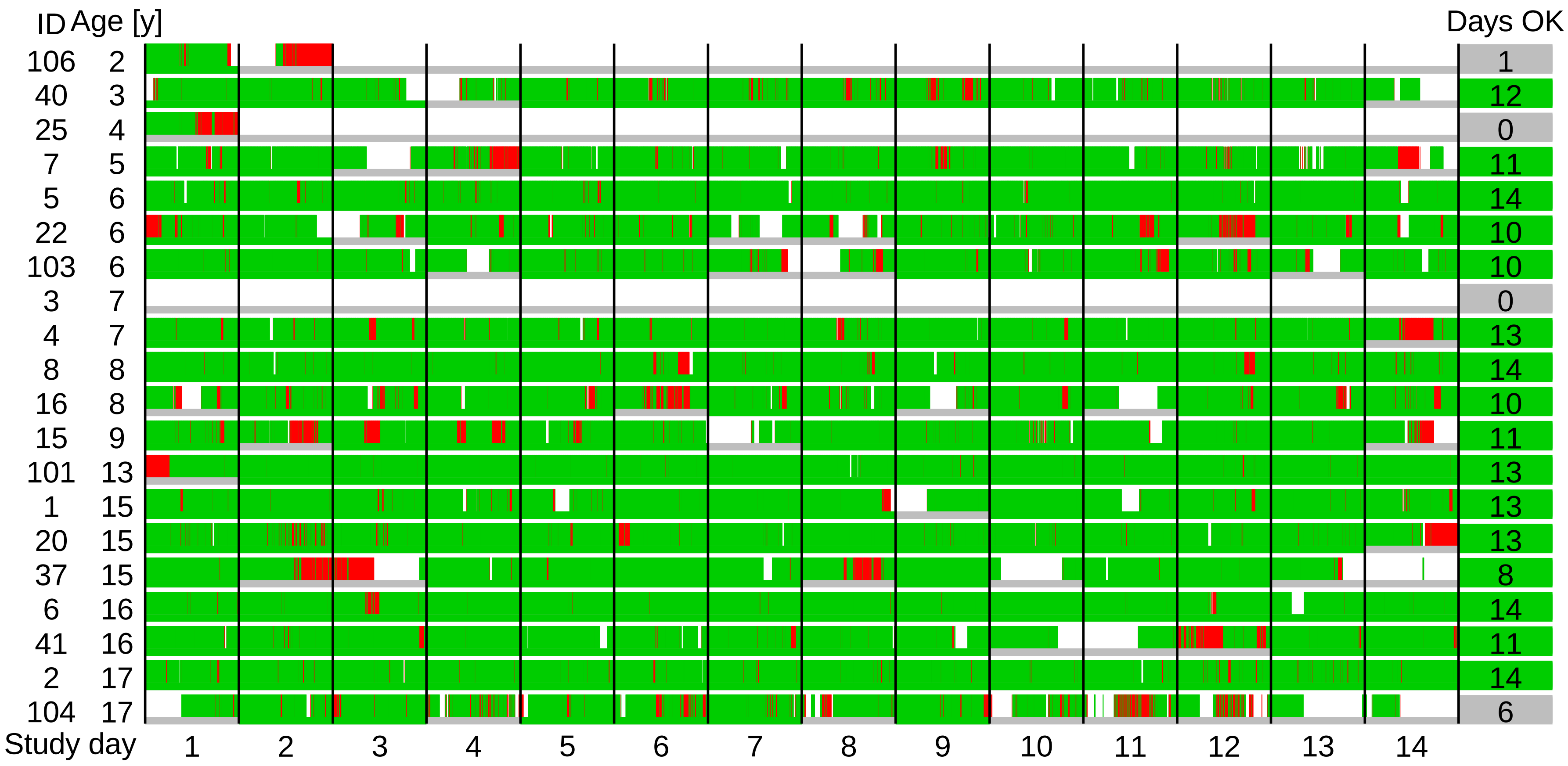

Displayed are the results of heart rate recording, irrespective of transmission delays, by the Everion® in A) the predecessor study, NCT NCT04134429 (from ref. 10); and B, in this study.

Legend: green, at least sufficient data quality; red, poor data quality; white, no data recorded; gray, study terminated preterm; Days ≥18 h / Days OK, number of days with at least 18 hours of at least sufficient data quality per patient; ID, patient ID

Online Resource Figure S4 – All signals, all patients and all study days

A

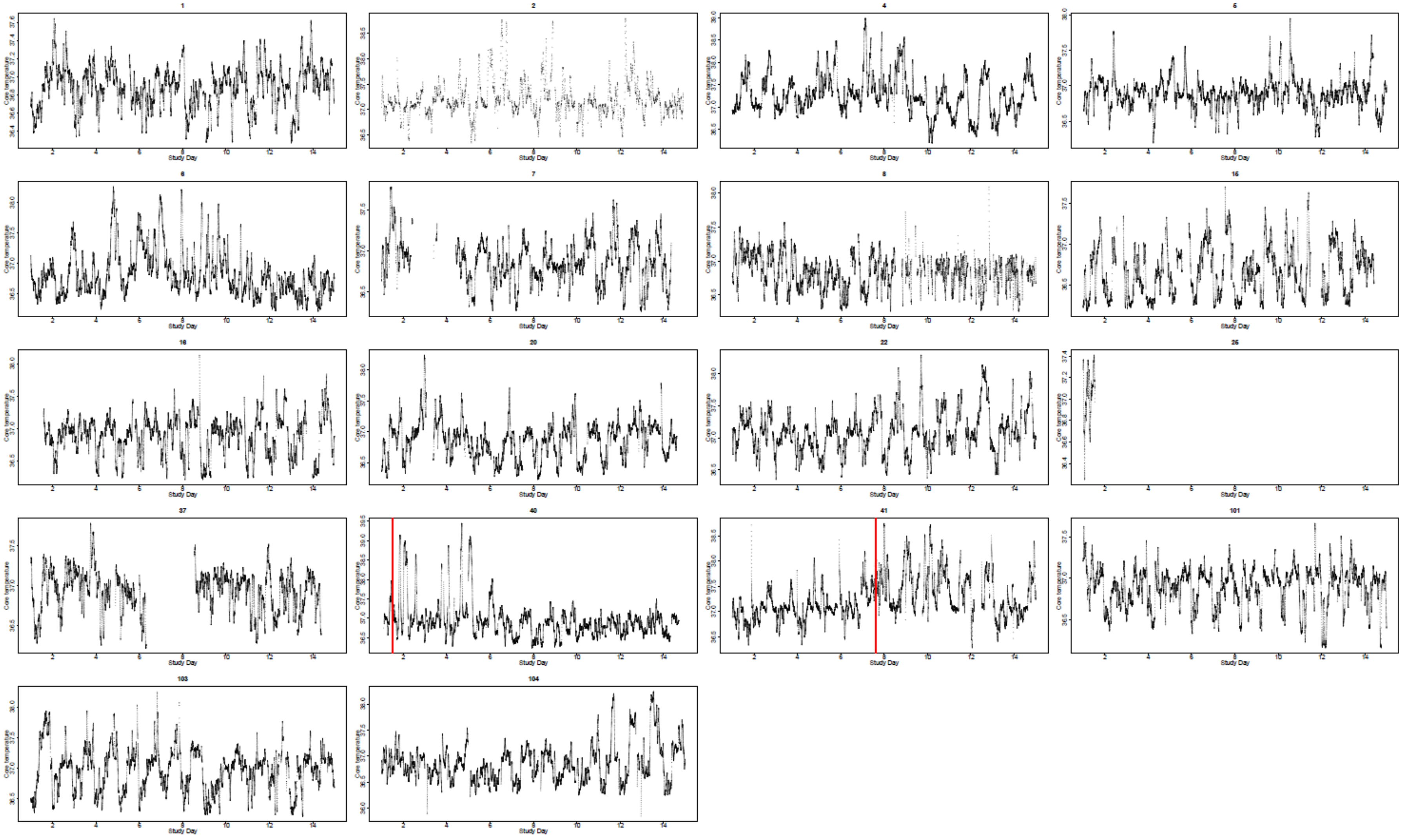

B

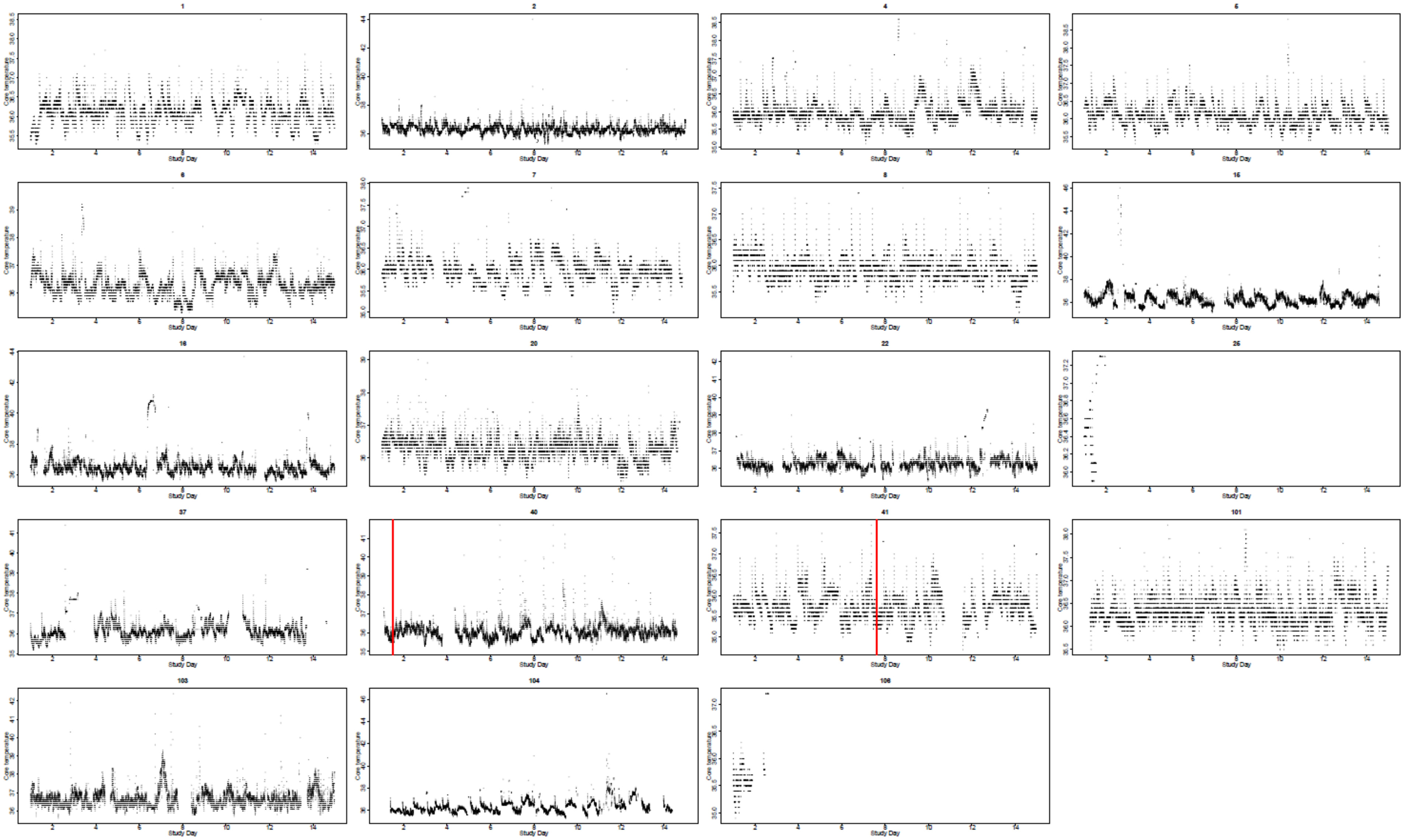

C

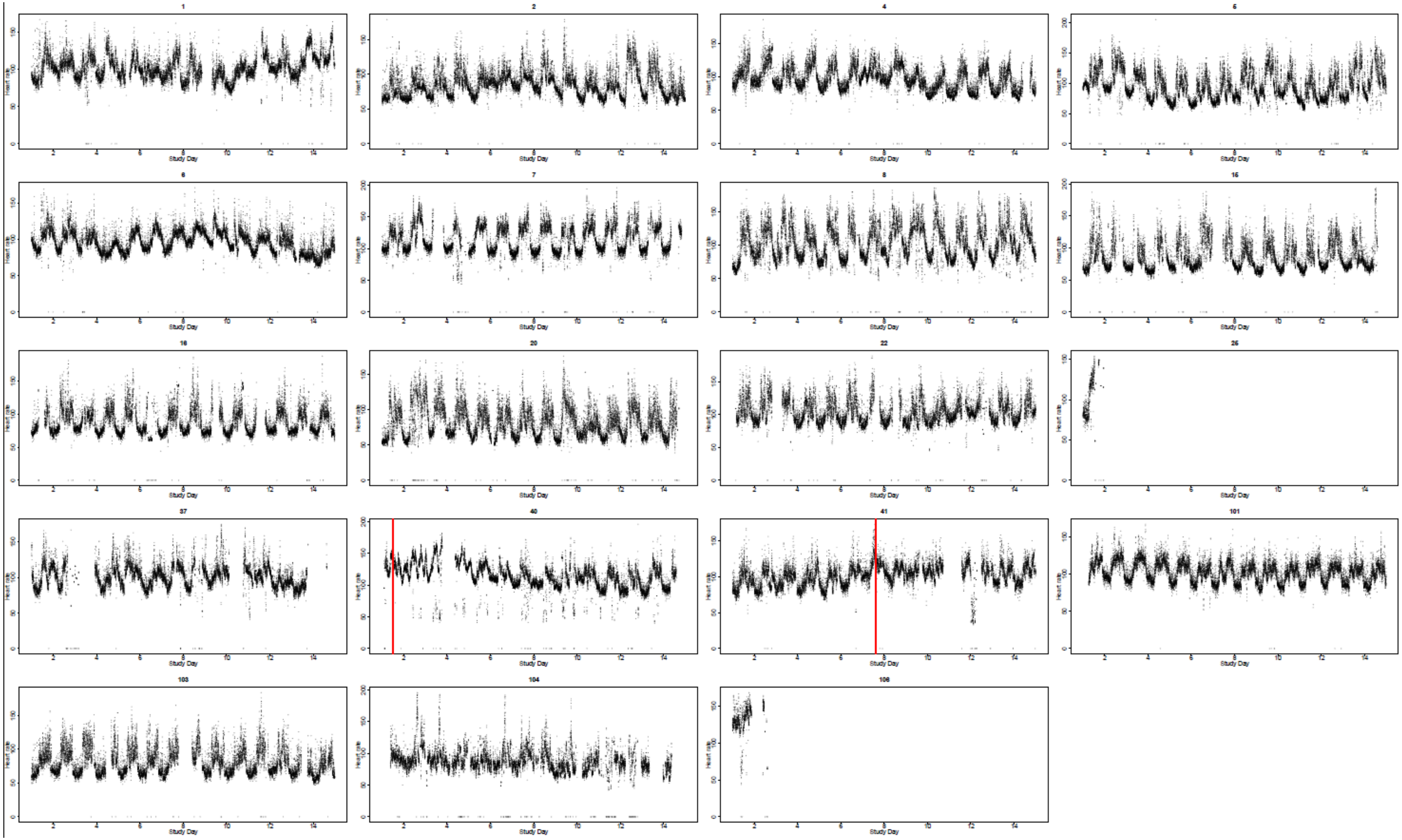

D

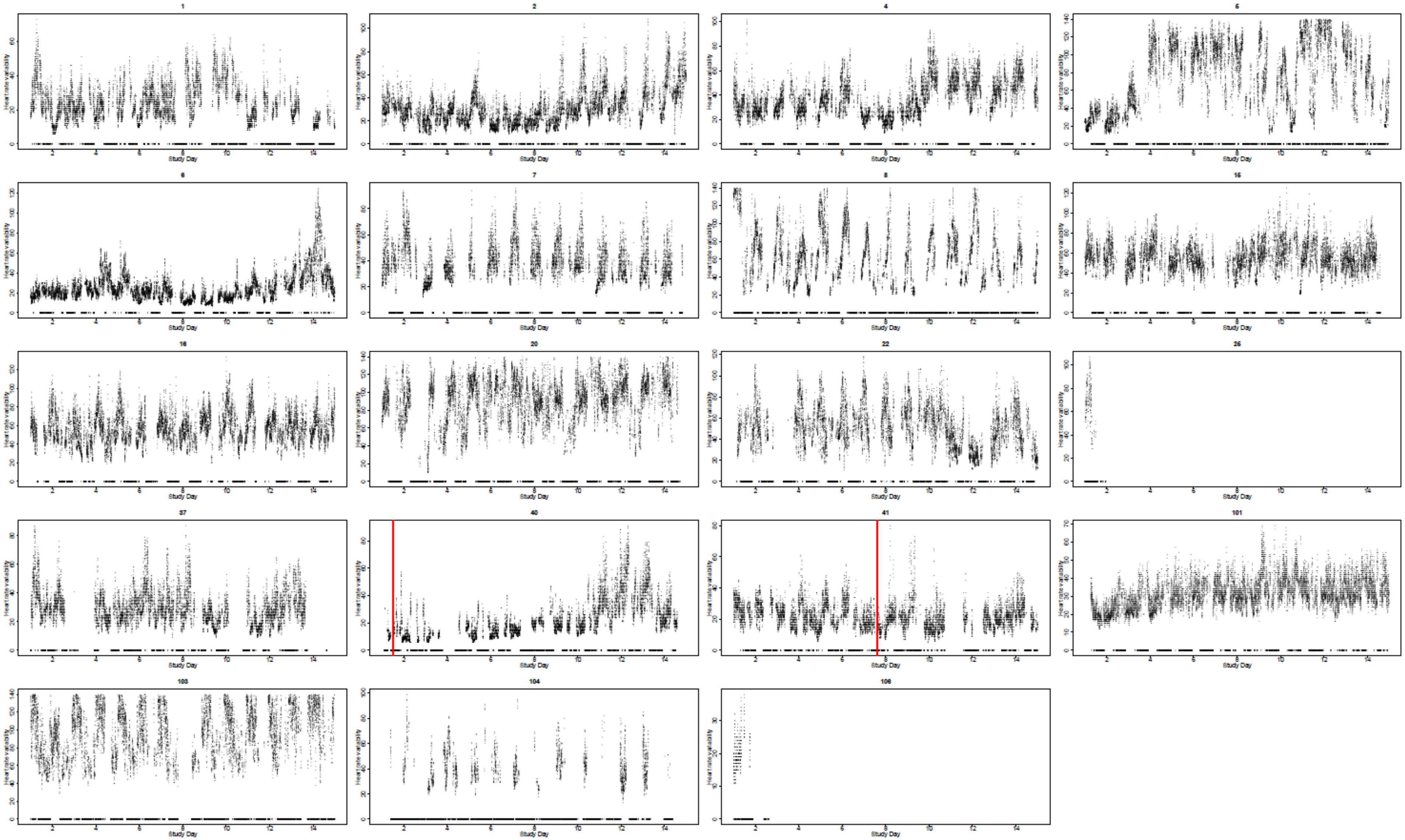
1
2
4
5
6
7
8
16
18
20
22
26
37
40
41
101
103
104
106
Heart rate variability
Study Day

Displayed are the results of all participants during the 14 days study period: Core temperature measurements by the CORE® in A) and by the Everion® in B), hear rate in C) and heart rate variability in D) measured by the Everion® in good quality (score ≥50). Red lines: Event with fever or infection.

Legend: x-Axis: Study day, and y-Axis: Height of measured signal. Number: Patient ID.

Online Resource Figure S5 – Transmission delays of CORE® and Everion® data

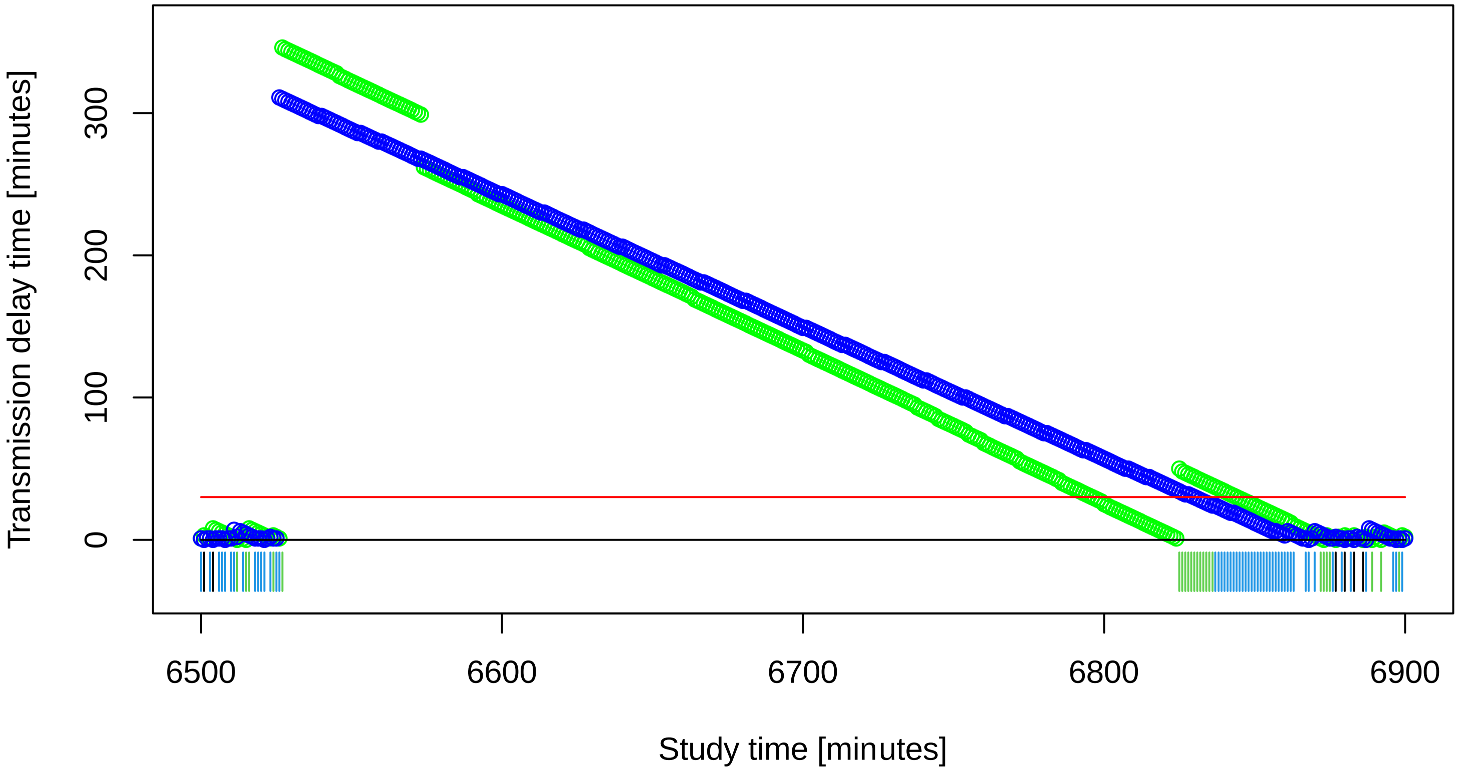

Displayed are transmission delay time (on the Y-axis, in minutes) versus study time (on the X-axis, in minutes) of the CORE® (green circles) and the Everion® (blue circles) in patient 8 during part of study day 5. The delays have originated in this section of study time because neither WD transmitted during 5 hours (white section below 0. Green lines below 0 indicate CORE® transmission, blue lines indicate Everion® transmission, black lines indicate CORE® and Everion® transmission in the same minute).

Online Resource Figure S6 – Data volume measurement in a section with non-delayed data transmission for CORE® and Everion®

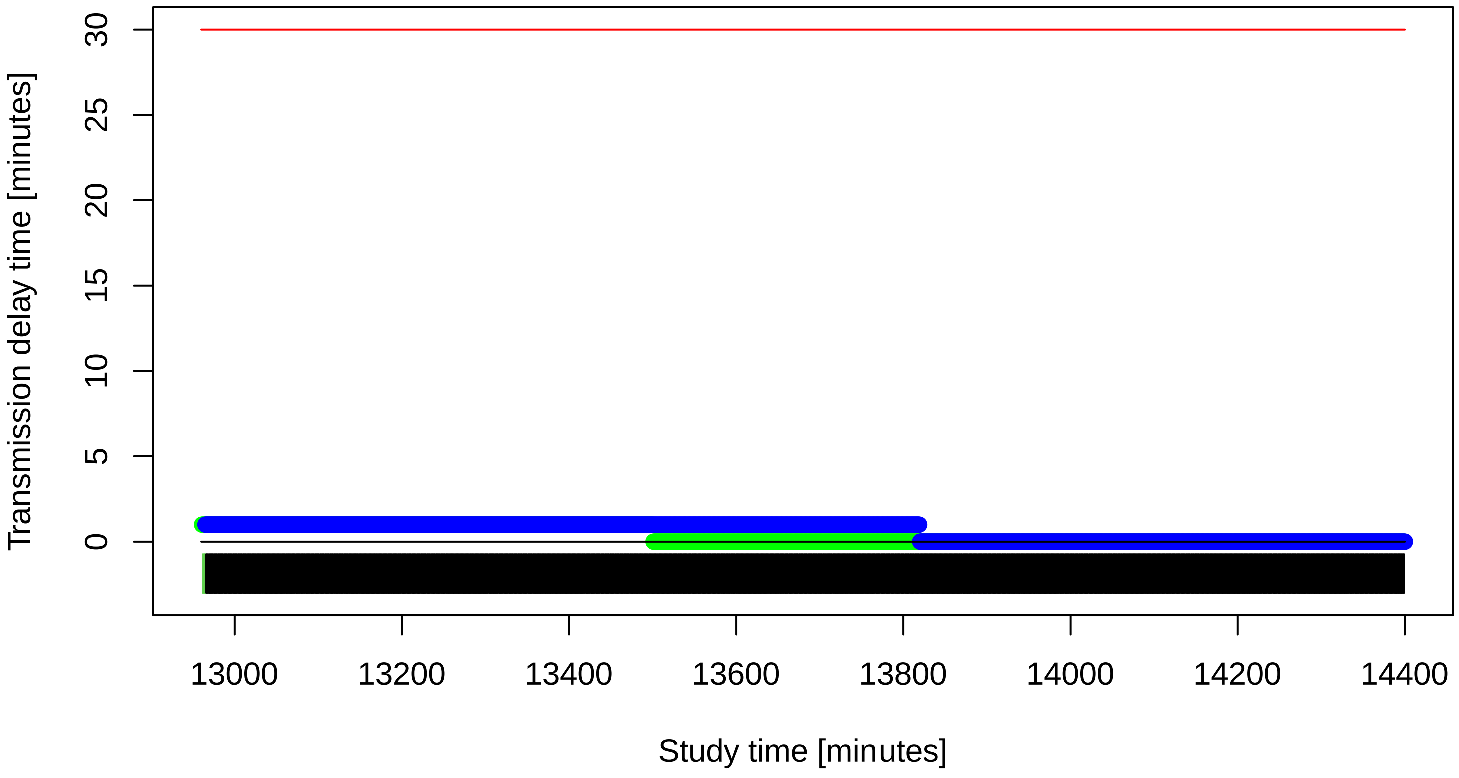

Displayed are transmission delay time (on the Y-axis, in minutes) versus study time (on the X-axis, in minutes) of the CORE® (green circles, forming a thick green line here, partially hidden by the Everion® circles/line) and of the Everion® (blue circles, forming a thick blue line here) in patient 37 on nearly the entire study day 9. The horizontal red line indicates 30 minutes delay, the limit between timely and delayed transmission. The black lines below 0, forming a black band here, indicate that both the CORE® and the Everion® transmitted in the same minute.

Online Resource Figure S7– Transmission speed measurement in a section with continuous transmission of delayed CORE® data

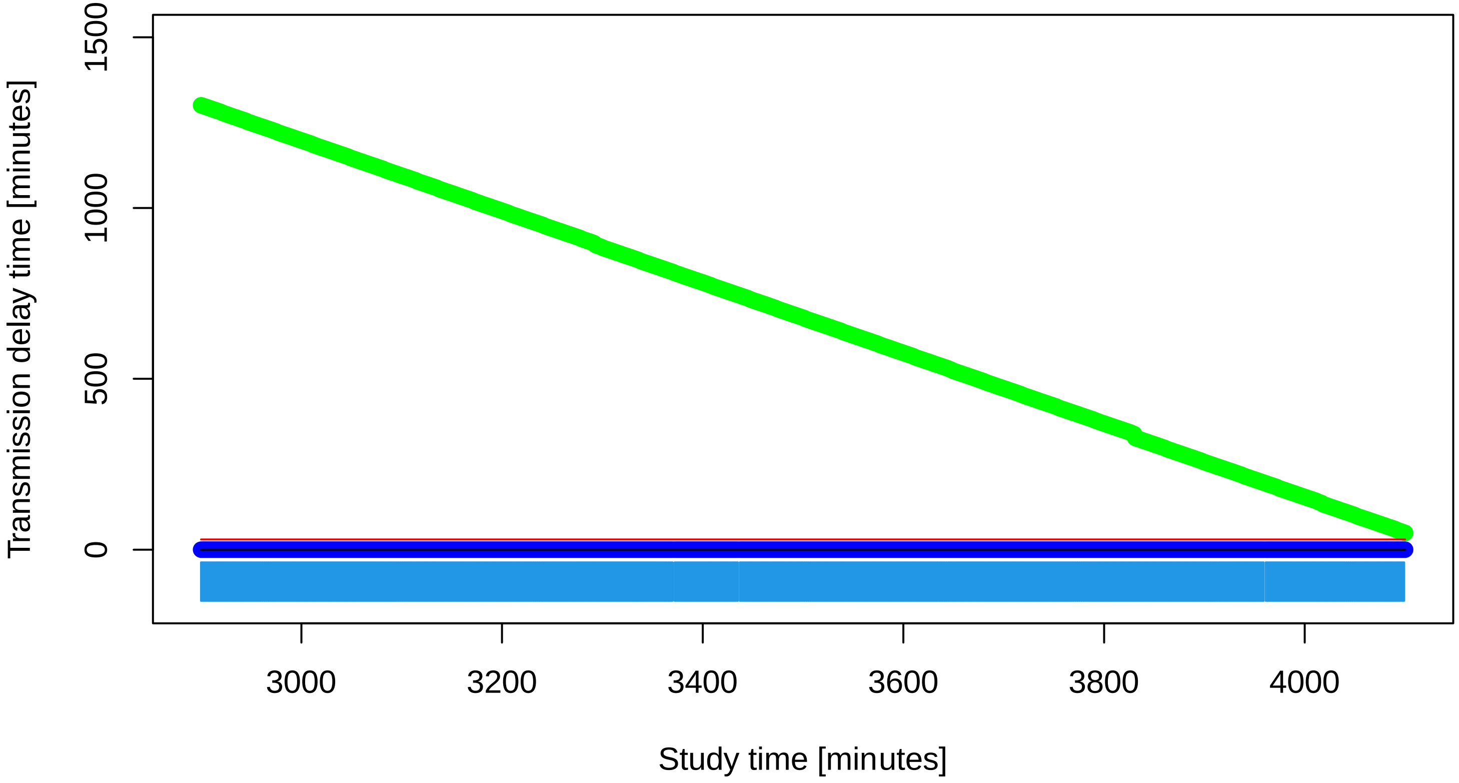

Displayed are transmission delay time (on the Y-axis, in minutes) versus study time (on the X-axis, in minutes) of the CORE® (green circles, forming a thick green line here) in patient 1 on nearly the entire study day 3. The delay has originated in this section of study time because the Everion® was transmitting exclusively (blue lines below 0, forming a blue band here). Transmission of the CORE® is beyond the right margin of the figure, and thus not visible here. The horizontal red line indicates 30 minutes delay, the limit between timely and delayed transmission.

Online Resource Figure S8 – Transmission speed measurement in a section with continuous transmission of delayed Everion® data

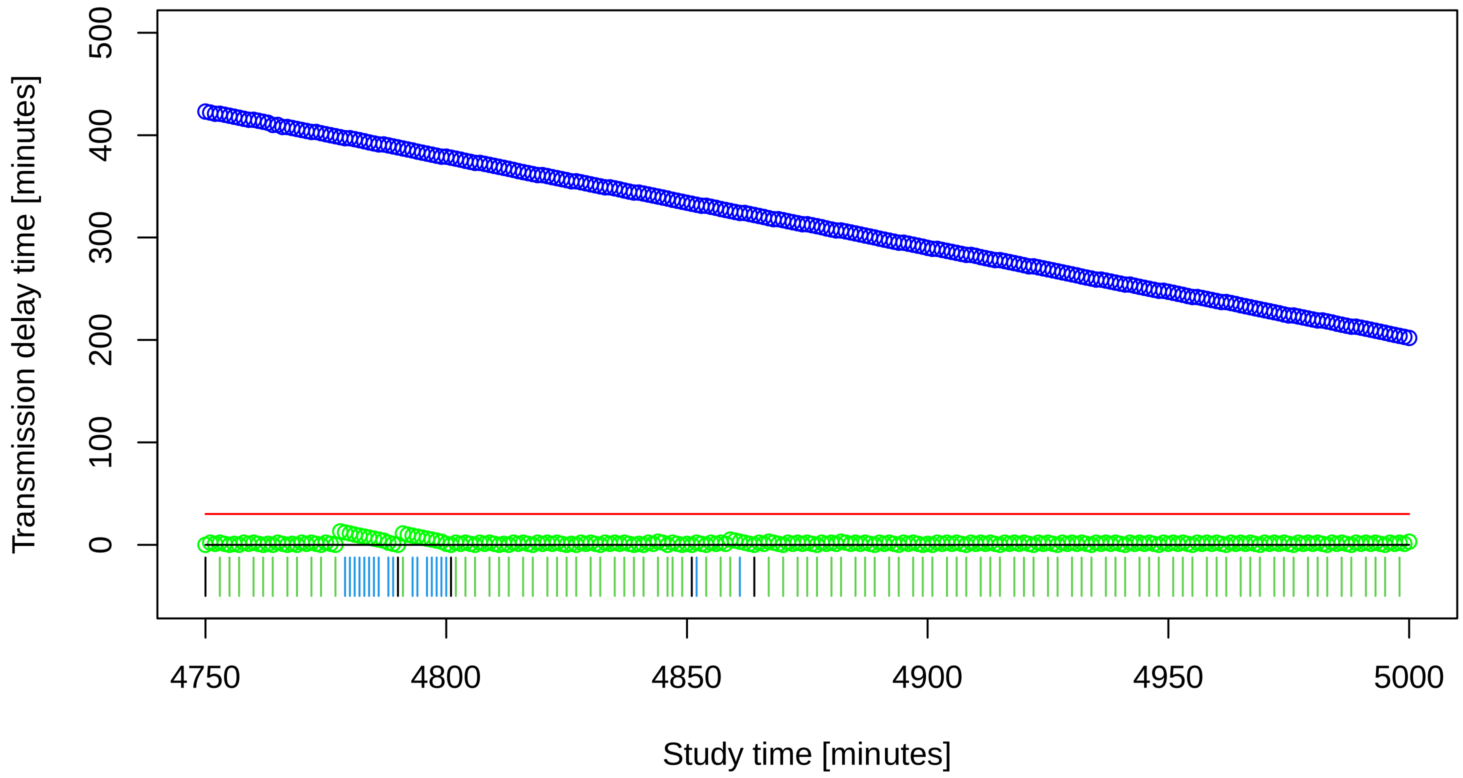

Displayed are transmission delay time (on the Y-axis, in minutes) versus study time (on the X-axis, in minutes) of the Everion® (blue circles) in patient 1 during part of study day 4. The delay has originated in this section of study time because the CORE® was transmitting nearly exclusively (green lines below 0; blue lines indicate Everion® transmission, black lines indicate CORE® and Everion® transmission in the same minute). Transmission of the Everion® is beyond the right margin of the figure, and thus not visible here. The horizontal red line indicates 30 minutes delay, the limit between timely and delayed transmission.

Online Resource Figure S9 – Transmission delays and simulated delays of CORE® and Everion® data

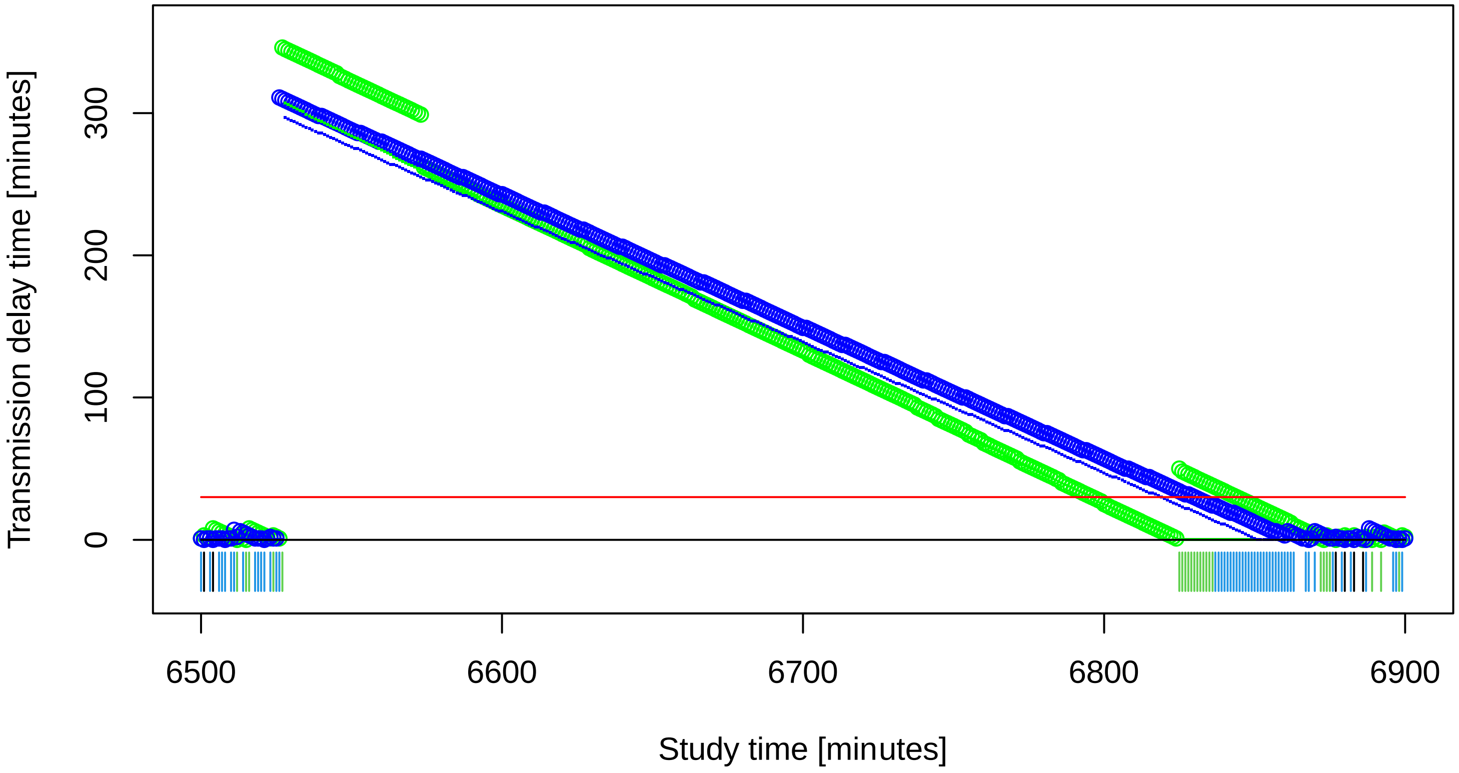

Displayed are transmission delay time (on the Y-axis, in minutes) versus study time (on the X-axis, in minutes) of the CORE® (green circles) and the Everion® (blue circles) in patient 8 during part of study day 5, plus simulated delays without transmission bottleneck (green and blue dots, respectively). Green lines below 0 indicate CORE® transmission, blue lines indicate Everion® transmission, black lines indicate CORE® and Everion® transmission in the same minute).

Online Resource Table S1 - Basic patient characteristics.

	Patients screened	Patients not asked for informed consent	Patients who refused informed consent	Patients in study
Number of patients	65[a]	28	10	20
Age [years; median (range)]	7 (4 to 14)	5 (2 to 9)	8 (4 to 14)	8 (6 to 15)
<6 years	25 (38%)	16 (57%)	4 (40%)	4 (20%)
Sex				
Female	32 (49%)	16 (57%)	6 (60%)	7 (35%)
Male	33 (51%)	12 (43%)	4 (40%)	13 (65%)
Type of malignancy				
Acute lymphoblastic leukemia	31 (48%)	13 (46%)	6 (60%)	9 (45%)
Other hematologic malignancies	5 (8%)	1 (4%)	1 (10%)	3 (15%)
Central nervous system tumor	10 (15%)	3 (11%)	2 (20%)	3 (15%)
Solid tumor	19 (29%)	11 (39%)	1 (10%)	5 (25%)

[a] Of 65 patients screened, seven did not meet inclusion criteria

Online Resource Table S2 - Details on patient and parent satisfaction and user-friendliness.

	CORE® (n= 19)[a]			Everion® (n= 19)[a]		
**Questions regarding the WDs**	**yes**	**sometimes**	**no**	**yes**	**sometimes**	**no**
The WD is suitable to record vital signs in children undergoing chemotherapy for cancer	18 (95%)	0 (0%)	1 (5%)	17 (89%)	0 (0%)	2 (11%)
The WD is easy to use	16 (84%)	2 (11%)	1 (16%)	15 (79%)	0 (0%)	4 (21%)
The WD is comfortable to wear	19 (100%)	0 (0%)	0 (0%)	13 (68%)	4 (21%)	1 (5%)
The WD is too big	0 (0%)	0 (0%)	19 (100%)	4 (21%)	1 (5%)	14 (74%)
The WD is too heavy	0 (0%)	0 (0%)	19 (100%)	1 (5%)	17 (89%)	1 (5%)
The WD slips occasionally	4 (21%)	3 (16%)	12 (63%)	4 (21%)	4 (21%)	11 (58%)
The WD is easy to charge	16	1 (5%)	2 (11%)	18 (95)	0 (0%)	1 (5%)
The time needed to use the WD too long	2 (11%)	2 (11%)	15 (79%)	2 (11%)	1 (5%)	16 (84%)
**Questions regarding the Gateway (n=19)**	**yes**	**sometimes**	**no**			
The gateway is easy to start	16 (84%)	0 (0%)	2 (11%)			
The gateway is too heavy	7 (38%)	3 (16%)	8 (42%)			
The gateway is tedious to carry	13 (68%)	3 (16%)	3 (16%)			
The gateway is getting too hot	8 (42%)	1 (5%)	9 (47%)			
The gateway is tedious to charge	0 (0%)	1 (5%)	18 (95%)			
The battery life of the gateway is enough	17 (89%)	0 (0%)	1 (5%)			
**Questions regarding data access and daily feedbacks (n=19)**	**yes**	**sometimes**	**no**			
Data access worked fine	16 (84%)	2 (11%)	0 (0%)			
Data access was motivating	11 (58%)	3 (16%)	2 (11%)			
Data access lead to insecurity	3 (16%)	4 (21%)	11 (58%)			
The data shown was enough	12 (63%)	3 (16%)	2 (11%)			
Daily feedback was motivating	11 (58%)	3 (16%)	2 (11%)			
Daily feedback was disturbing	0 (0%)	0 (0%)	19 (100%)			

[a] numbers do not always add up to 19 as not all participants answered all the questions.

Online Resource Table S3 - Details on feedbacks, additional contacts and problems identified.

**Daily feedback** [a]	**241**
Text message	232
Call	6
E-mail	1
Unclear	2
**Feedbacks given** [b]	
Everything ok	164
No data transferred	20
Data transferred but with delay	27
Other [c]	65
Everion®	
- Reminder to charge or switch the WD	6
- Reminder to put the WD closer to the gateway	19
CORE®	
- Reminder to charge the WD	15
- Quality problems	3
**Additional contacts**	40 (on 36 days, max 2 additional contacts)
Reasons:	
Missing data	14
Other [c]	26
- questions how to use the WDs	6
- Information about the use of the WDs by participants	11
- asking for a new link to access the data	1
- problems with data transfer	2
**Problems identified**	
Non-wearing	3
Missed charging of the WD	5
Missed charging of the gateway	4
Gateway out of range	5
Unclear problem with the gateway	5
WD broken	2
WD fell off during the night	1

[a] no feedback given to one hospitalized participant and to participant number 106 on the day of study drop out, [b] more than one option possible, [c] not all specified.

**Online Resource Table S4 –** Signals recorded by Everion® and CORE®.			
**Everion®**	**unit**	**range**	**Quality Score**
E.1 Core temperature	°C	21-46	EQ.1
E.2 Heart rate	bpm	30-240	EQ.2
E.3 Heart rate variability	ms	0-255ms	EQ.3
E.4 Respiration rate	bps	2-60	EQ.4
E.5 Skin temperature	°C	0-60	-
E.6 Perfusion index	-	0-5	-
E.7 Health score	-	0-100%	-
E.8 GSR (Galvanic skin response)	kOhm	0-5	-
E.9 Motion-activity	-	0-255	-
E.10 Energy expenditure	Cal/s	0-65535	EQ.10
E.11 Barometer-pressure	mbar	860-1060	-
E.12 Blood-pulse-wave	-	0-5.1	-
E. 13 Number-of-steps	steps	0-65535	-
E. 14 Activity classification	Motion classified from the range	0-21	EQ.14
E.15 Training_effect_score	percent	0-100%	-
E.16 Activity_score	percent	0-100%	-
E.17 Relax_stress_intensity_score	percent	0-100%	-
E.18 Sleep_quality_index_score	percent	0-100%	-
**CORE®**	**unit**	**range**	**Quality Score**
C.1 Core temperature	°C		CQ.1
C.2 Skin temperature	°C		-
